# *N*-Glycoengineering of insect cells for tri-antennary *N*-glycan biosynthesis

**DOI:** 10.1038/s41598-026-41152-8

**Published:** 2026-03-16

**Authors:** Hiroyuki Kajiura, Naokuni Nishiguchi, Reimi Lai Sang Sawada-Choi, Yuto Sana, Ryo Misaki, Kazuhito Fujiyama

**Affiliations:** 1 International Center for Biotechnology, The University of Osaka, 2-1 Yamada-oka, Suita, Osaka 565-0871 Japan; 2 Institute for Open and Transdisciplinary Research Initiatives (OTRI), The University of Osaka, 2-1 Yamada-oka, Suita-shi, Osaka 565-0871 Japan; 3 Department of Biotechnology, Graduate School of Engineering, The University of Osaka, 2-1 Yamada-oka, Suita, Osaka 565-0871 Japan; 4 The University of Osaka Cooperative Research Station in Southeast Asia (OU:CRS), Faculty of Science, Mahidol University, Bangkok, Thailand

**Keywords:** *N*-Glycosylation, Insect cells, *N*-Acetylglucosaminyltransferase IV, *N*-Acetylhexosaminidase, GlcNAc extension, Glycosylation, Glycobiology

## Abstract

Insect cells are attractive hosts for biopharmaceutical production due to their high productivity and mammalian-like post-translational modifications. However, their insect-specific *N*-glycans differ from mammalian types, thereby reducing product desirability. Here, with an emphasis on engineering *N*-glycosylation in insect cells, we aimed to develop a more tractable *Spodoptera frugiperda* Sf9 cell platform as a practical alternative to insect-based systems for engineering the production of tri-antennary *N*-glycans. A database search revealed that silkworm possesses *N*-acetylglucosaminyltransferase IV (GNTIV), a putative glycosyltransferase essential for tri-antennary *N*-glycan synthesis; however, it was not functional in vitro. Then, human GNTIV was introduced into insect cells, resulting in the production of small amounts of tri-antennary *N*-glycans. This suggested the need for additional factors to efficiently biosynthesize tri-antennary *N*-glycans. Subsequently, additional insect-derived glycosyltransferases, such as active GNTI and/or GNTII, were co-expressed with GNTIV. Co-expression of three *N*-acetylglucosaminyltransferases effectively led to the increased biosynthesis of tri-antennary *N*-glycans. On the other hand, trimming of the *N*-glycan structure was also observed due to the action of one or more endogenous glycosylhydrolases, which hydrolyze the terminal *N*-acetylglucosamine residue in insect cells. These facts indicate that effective tri-antennary *N*-glycan biosynthesis in insect cells requires not only the introduction of exogenous glycosyltransferases but also the knockdown or knockout of endogenous glycosylhydrolase(s).

**Supplementary Information:**

The online version contains supplementary material available at 10.1038/s41598-026-41152-8.

## Introduction

Biopharmaceuticals exhibit high activity and effectiveness against diseases requiring specific clinical treatment, without the potential side effects associated with small-molecule drug treatment^1^. Using heterologous hosts for the production of valuable proteins, particularly for medical or veterinary purposes, could offer an alternative system to mammalian cells, which currently serve as the primary host for biopharmaceutical protein production. This approach is desired as a next-generation protein production platform. Each production host has advantages, including factors such as production cost, time, scalability, and yield^2^. In particular, post-translational modifications directly contributing to protein function in vivo are important and essential factors for the production of pharmaceutical proteins that could be applied to mammals, including humans. A key post-translational modification is *N*-glycosylation. *N*-Glycosylation, involving the attachment of an oligosaccharide to Asn residue within the consensus sequence Asn-X-Ser/Thr (excluding X for Pro), is highly conserved in eukaryotes. It is estimated that more than half of proteins in nature are synthesized as *N*-glycoproteins^3^. This is also true for pharmaceutical proteins, as they require *N*-glycosylation for bioactivity. However, *N*-glycosylation is not identical in each eukaryote and has species specificity^4,5^. This species-specific *N*-glycosylation leads to the biosynthesis of structurally different *N*-glycans on proteins. This could be a disadvantage in terms of heterologous protein production due to the decrease in half-life in the circulation and the potential for immunogenicity due to differences in the sugar residues comprising the *N*-glycans. This makes the insect-producing biopharmaceutical protein less preferable. Therefore, the remodeling of the species-specific *N*-glycosylation machinery into mammalian type should be mandatory for the therapeutic use of the *N*-glycoproteins produced in heterologous expression systems for pharmaceutical purposes.

The modification of *N*-glycosylation machinery, called *glyco-engineering*, enabled the biosynthesis of mammalian-type *N*-glycan in plants, yeasts, and insects^6–12^. In most cases, “mammalian-type *N*-glycan” refers to bi-antennary *N*-glycans with galactose (Gal) and especially *N*-acetylneuraminic acid (NeuAc) residues at the non-reducing terminal of *N*-glycan. In mammals, complex-type *N*-glycans also consist of a core α1,6-fucose (α1,6-Fuc) residue and branched residues including bisecting *N*-acetylglucosamine (GlcNAc). These sugar residues can either enhance or inhibit the antibody-dependent cellular cytotoxicity activities of IgG^13–16^. Thus, it is desired to produce pharmaceutical proteins with optimal *N*-glycan structures. It is therefore necessary to establish various platforms to produce *N*-glycan-optimized pharmaceuticals by modifying the *N*-glycosylation pathway.

Besides Gal and NeuAc residue(s), α1,6-fucosylation, and bisecting GlcNAc, branched *N*-glycans are also characteristic of mammalian-type *N*-glycans^17^. Branched *N*-glycans are synthesized by a series of *N*-acetylglucosaminyltransferases^18^. Among them, *N*-acetylglucosaminyltransferase IV (GNTIV) is a glycosyltransferase categorized into GT54 protein in CAZy (http://www.cazy.org/), which catalyzes one of the initial transfers of GlcNAc from the activated sugar nucleotide of UDP-GlcNAc to *N*-glycan to synthesize tri-antennary *N*-glycans. This tri-antennary *N*-glycan structure formed by GNTIV plays important roles in vivo. Mice deficient in GNTIV showed signs of metabolic diseases, such as type 2 diabetes and reduced insulin activity^18,19^. Tri-antennary *N*-glycans might serve as ligands to receptors, facilitating the binding of carrier proteins to receptors, whereas tri-antennary *N*-glycans might also act as steric hindrance to receptor binding, reducing binding affinity. For example, the branched *N*-glycans on erythropoietin (EPO) enhance binding to the erythropoietin receptor in vitro, but in vivo, the large structure of the branched *N*-glycans contributes to the delay of EPO clearance from the circulation, resulting in prolonged erythropoiesis^20^. This is attributed to the effect of increased terminal NeuAc content in EPO *N*-glycans, resulting in improved in vivo biological activities^21^. These facts suggest that GNTIV is also essential for glyco-engineering of *N*-glycan in a heterologous system and becomes a target enzyme that should function in the newly established *N*-glycosylation pathway. Previously, heterologous expression of GNTIV in plants was performed and tri-antennary *N*-glycans were successfully synthesized^8,22,23^. However, this approach has not yet been examined in yeasts and insects including insect cells. Insects predominantly synthesize high-mannose and paucimannose *N*-glycans, and their structural profiles vary extensively across species^24–26^. In addition, insect-specific modifications, particularly core α1,3- and/or α1,6-fucosylation, represent unique *N*-glycan structures that are critically involved in biological processes such as immune response, wing formation, and nervous system development^27–29^. Previous studies have further demonstrated that *N*-glycosylation in insects is indispensable for normal development and viability. Disruption of *N*-glycan attachment or processing in insects results in severe developmental defects, including impaired metamorphosis, appendage malformation, and increased mortality^30,31^, and in more severe cases of defective *N*-glycan modifications, lethality has also been reported^28^. Together, these insights highlight that precise control of *N*-glycosylation is tightly linked to insect growth and viability. Importantly, the unique *N*-glycan structures found in insects are not only key determinants of insect physiology but also represent critical targets for *N*-glycan engineering when producing recombinant therapeutic *N*-glycoproteins in insect-based expression systems, where mammalian-compatible *N*-glycan profiles are required.

Recently, the *N*-glycosylation pathway in insects has been vigorously analyzed and modified^32–38^. Insects meet the criteria for serving as “*protein factories*” that produce useful proteins, including pharmaceutical proteins, with high productivity. Especially, the silkworm *Bombyx mori* is considered an excellent host because of its high productivity, genome information availability, and established genetic engineering technologies^39–41^. *B. mori* possesses mainly insect-type *N*-glycans with a pauci-mannose type which are composed of only a few mannose residues remaining on the core structure (typically Man_1–4_GlcNAc_2_Fuc_0–2_) and are commonly found in invertebrates, such as insects and nematodes^42^. However, as in other cases, *N*-glycan heterogeneities were observed depending on the stage and organ in *B. mori*. Interestingly, we demonstrated that the *B. mori* middle silk gland (MSG), which contributes to the secretion of fibroin (a major part of the silk fiber), accumulated bi-antennary *N*-glycans (GlcNAc_2_Man_3_GlcNAc_2_, GN2M3; Man: mannose) (Supplementary Fig. S1 online) essential for mammalian-type *N*-glycans^43^. Furthermore, *B. mori* has a putative GNTIV homologue in the genome, and its carbohydrate-binding modules have been characterized^44^. Thus, the utilization of *B. mori* MSG and of the gene for *N*-glycoengineering could be fully expected to modify the *N*-glycan structure into the mammalian type of branched *N*-glycan structures in insects. A recent study also suggests that insects could produce recombinant *N*-glycoproteins with branched *N*-glycans^45^. However, that study focused only on a single model protein, for which mammalian-derived glycosyltransferase genes essential for branched *N*-glycans were introduced. Consequently, the general applicability of this approach across insect species, particularly Lepidoptera, remains unclear. Previous studies provide evidence that tri-antennary *N*-glycans are not detected in Lepidoptera or in recombinant proteins from these species^25,46^, whereas such structures have been reported in Diptera^47,48^ and Hymenoptera^26^. Thus, further confirmation of the applicability of this successful *N*-glycosylation to all *N*-glycoproteins is still needed. In particular, for lepidopteran species such as *B. mori*, achieving the biosynthesis of branched *N*-glycans would likely require a systematic assessment of their intrinsic potential to generate such complex structures, followed by rational glyco-engineering strategies, including the introduction of appropriate glycosyltransferase(s) if necessary.

**Fig. 1:**
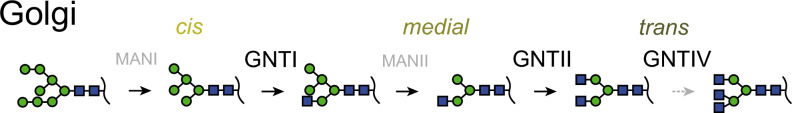
*N*-Acetylglucosamine transfer reactions engineered in this study. Enzymes shown in black indicate overexpressed enzymes, whereas enzymes shown in gray represent endogenous enzymes.

To achieve the biosynthesis of mammalian-type *N*-glycans in insect cells, we focused on tri-antennary *N*-glycan biosynthesis (Fig. 1). In this study, expression of various combinations of *B. mori* glycosyltransferases and the effects on *N*-glycosylation remodeling were first examined in *Spodoptera frugiperda* Sf9 cells by mimicking *B. mori*, enabling a quicker and higher-throughput analysis compared to *B. mori* larvae. This study assessed the GNTIV homolog in *B. mori* and explored the effect of *N*-acetylglucosaminyltransferases that contribute to the process, suggesting the potential future applications.

## Results

### Silkworm has a nonfunctional GNTIV-like protein

In the context of insect *N*-glycosylation remodeling through exogenous gene expression, genes sourced from insect species are deemed ideal and preferable over those from other species such as mammals. To express the insect-derived *GNTIV* gene in Sf9 cells, a database search using the Silkworm Genome Informative Database (SGID, http://sgid.popgenetics.net/) and GNTIV-A, a human GNTIV (hGNTIV) homologue, as a query revealed the evidence that the silkworm *Bombyx mori* had a GNTIV homologue, gene code KWMTBOMO00036, in the genome. The putative *B. mori* GNTIV-like protein, characterized by a longer polypeptide and exhibiting higher similarity compared to the sequence in SGID, was also deposited in the KEGG database (https://www.genome.jp/kegg/) (Supplementary Fig. S2 online). The *B. mori* GNTIV-like protein in KEGG was termed BmGNTIV and analyzed in further research. BmGNTIV is a protein of 596 amino acids with a calculated molecular mass of 69.7 kDa; it possesses two potential *N*-glycosylation sites. The amino acid sequence similarities between BmGNTIV and human hGNTIVa and hGNTIVb were 30.8% and 31.9%, respectively. The alignment revealed that N-terminal region had low similarities to each other, whereas BmGNTIV and human GNTIVs exhibited more conserved amino acid regions from the putative catalytic domain to the C-terminus compared to the *N*-terminus (Supplementary Fig. S2 online). This was also supported by a recent finding that GNTIVs have C-terminal carbohydrate-binding modules^44^. Although these facts suggest the possibility that these conserved domains are likely essential for GNTIV activity, the activity of putative BmGNTIV activity has not been determined.

To verify BmGNTIV activity, putative BmGNTIV was expressed in Sf9 cells using the baculovirus expression vector system (BEVS) with a C-terminal His-tag sequence. This allowed for the production of BmGNTIV as a membrane-bounded and *N*-glycosylated protein (Supplementary Fig. S3a online). The purified BmGNTIV was successfully prepared, and the activity was examined in the presence of Mn^2+^ using UDP-GlcNAc and the PA-labeled sugar chain GlcNAc_2_Man_3_GlcNAc_2_ (GN2M3-PA) as donor and acceptor substrates, respectively. The reaction product was analyzed by RP-HPLC. However, no tri-antennary PA-sugar chain of GN3M3 was detected (Supplementary Fig. S3b online). To detect GNTIV activity, a variety of conditions were examined, including pH, buffer, temperature, and ion dependency were examined, but no GlcNAc transfer activity could be confirmed under any conditions (Supplementary Fig. S4 online). These results indicated that silkworm possesses GNTIV homologue with no native GlcNAc transfer activity.

### Expression of human GNTIV was functional in Sf9 cells but less effective for tri-antennary *N*-glycan synthesis

As synthesizing tri-antennary *N*-glycans using BmGNTIV was deemed unsuccessful, an alternative GNTIV isoform was considered. While other insect-derived GNTIV candidates, such as those found in honeybees, black soldier flies, stick insects, and *Drosophila*, were classified as GT54 proteins in CAZy, their activity had not been confirmed except for mammalian GNTIVs. Therefore, human GNTIVa was chosen for the biosynthesis of tri-antennary *N*-glycan in insect cells.

**Fig. 2:**
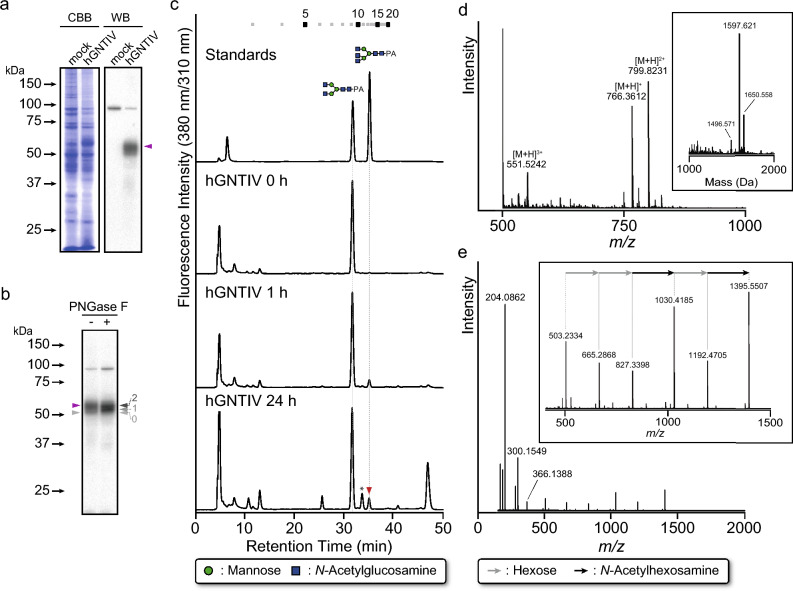
Analysis of hGNTIV expression and its reaction product. (**a**) CBB staining and Western blotting analysis of cell lysate of hGNTIV-expressing Sf9 cells. Anti-GNTIV antibody was used as a primary antibody. Triangles indicate hGNTIV expressed in Sf9 cells. (**b**) De-glycosylation analysis of hGNTIV expressed in Sf9 cells. Purple and gray triangles indicate the forms of native *N*-glycosylated and de-glycosylated hGNTIV, respectively. (**c**) RP-HPLC analysis of the reaction products. hGNTIV reaction was carried out using cell lysate, UDP-GlcNAc, and GN2M3 as a donor and an acceptor substrate, respectively. The elution position of the product was compared with authentic PA-sugar chains. Numbers at the top represent the elution positions of glucose units on the basis of the elution times of PA-isomalto-oligosaccharides with degrees of polymerization from 3 to 20. Green circles and blue boxes indicate Man and GlcNAc, respectively. Asterisk represents a peak that does not correspond to an authentic PA-sugar chain. (**d**) Observed MS spectrum of the reaction product indicated by the red triangle in Fig. 1c. The peak corresponding to the PA-derived glycans was observed in the doubly-charged state. The inset shows the deconvoluted MS spectrum. (**e**) MS/MS analysis of the precursor ion (*m/z* 799.8) corresponding to the *N*-glycan structure detected in Fig. 1d. The inset displays an enlarged view of the *m/z* 400–1500 region. Gray and black arrows indicate fragment ions derived from hexose and *N*-acetylhexosamine residues, respectively.

hGNTIV was expressed in Sf9 cells using BEVS. De-Glycosylation of hGNTIV by peptide: *N*-Glycosidase F (PNGase F) digestion was consistent with the calculated molecular mass of weight (Fig. 2a and b), indicating that hGNTIV was expressed as an *N*-glycoprotein. Insect-produced hGNTIV exhibited GlcNAc transfer activity toward GN2M3, resulting in the formation of tri-antennary *N*-glycan (Fig. 2c). These results suggested that hGNTIV was expressed as an active enzyme in Sf9 cells and might contribute to the remodeling of the *N*-glycosylation pathway for tri-antennary *N*-glycan biosynthesis.

**Fig. 3:**
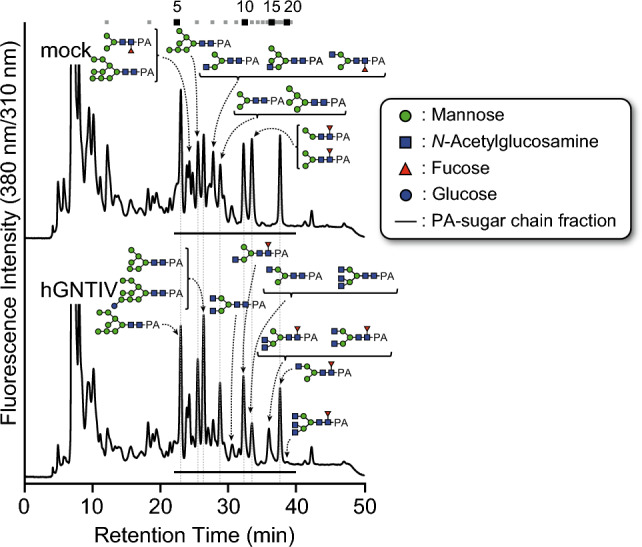
*N*-Glycan analysis of WT and hGNTIV-expressing Sf9 cells. Total soluble *N*-glycans prepared from *N*-glycoproteins and labeled with PA were analyzed by RP-HPLC with a C18 column. The major structures are shown in chromatographs. Underlining indicates the PA-labeled glycan fractions that were corrected and subsequently subjected to LC–MS/MS analysis for structural assignment. Numbers at the top represent the elution positions of glucose units.

To confirm the effect of hGNTIV expression on the remodeling of the *N*-glycosylation pathway, a comprehensive analysis of total *N*-glycans derived from soluble *N*-glycoproteins in hGNTIV-expressing Sf9 cells was conducted. Total soluble proteins were extracted from 5-day-post-infection Sf9 cells, followed by desalting, hydrazinolysis of *N*-glycoproteins, PA-labeling, and analysis by RP-HPLC and HPLC with tandem mass spectrometry (LC–MS/MS) (Fig. 3a, Supplementary Figure S5, S6, and Table S1 online). The ratios of *N*-glycan in WT Sf9 cells and hGNTIV-expressing Sf9 cells were similar to each other except for the ratio of M3FB structure; the ratio of M3FB in hGNTIV-expressing Sf9 cells became half that of the WT. On the other hand, the total *N*-glycan structure with terminal GlcNAc residue(s) increased from 2.7% in the WT to 10.5%. This *N*-glycan structure with terminal GlcNAc residue(s) included a tri-antennary *N*-glycan, such as GN3M3 and GN3M3F. Moreover, aberrant bi-antennary *N*-glycan GN2M3FC was also observed (Supplementary Figure S6), suggesting that hGNTIV functioned to produce *N*-glycans with β1,4-linked GlcNAc at GlcNAcβ1,2-Manα1,3-R. However, the total ratio of the tri-antennary *N*-glycans was 1.3%. Therefore, these results demonstrated that additional factors are required for the biosynthesis of tri-antennary *N*-glycan.

### Optimizing the expression of *N*-acetylglucosaminyltransferases increased *N*-glycans with terminal GlcNAc residues

A previous study demonstrated that overexpression of *N*-acetylglucosaminyltransferases contribute to a considerable enhancement of the biosynthesis of mammalian-type *N*-glycans^49^. The authors of that study hypothesized that overexpression of *N*-acetylglucosaminyltransferases might also contribute to tri-antennary *N*-glycan biosynthesis from the supply side of the acceptor substrate GN2M3. To enhance GlcNAc transfer activity in Sf9 cells, we focused on the recently characterized *B. mori* GNTI^43^ and GNTII-D^34^. In vitro activities of *B. mori* GNTI and GNTII-D, (bGNTI and bGNTII-D, respectively, hereafter) were determined, but the effects of overexpression of these two *N*-acetylglucosaminyltransferases on the remodeling of the *N*-glycosylation pathway in insect cells have not been examined.

**Fig. 4:**
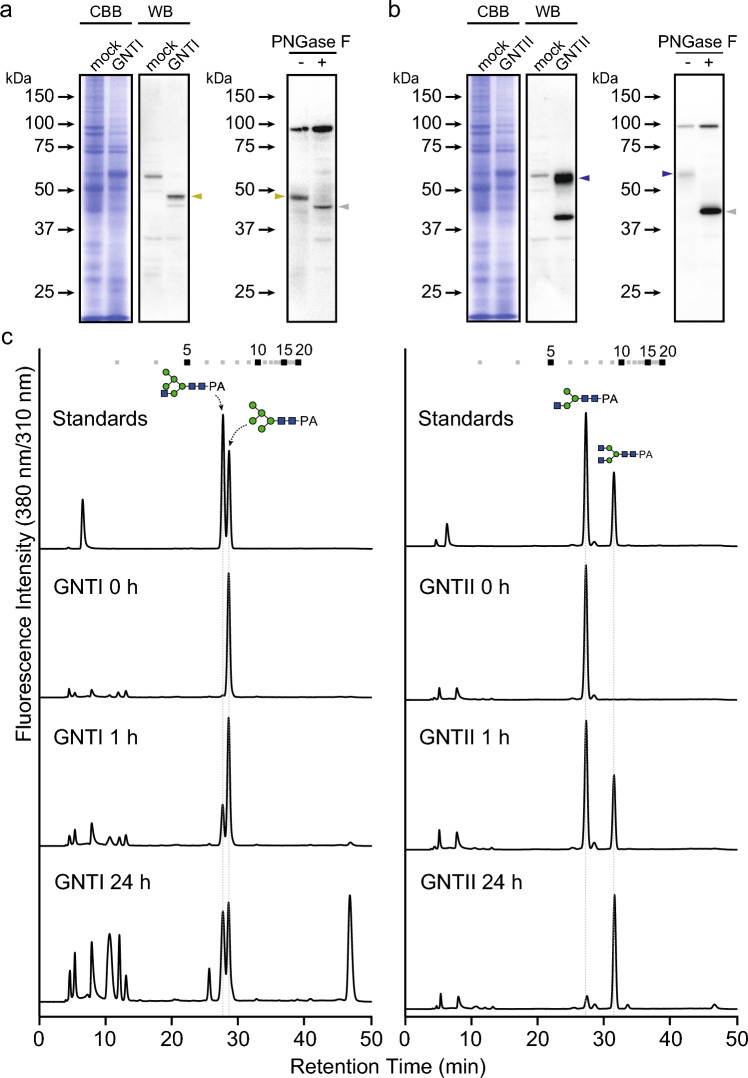
Analyses of bGNTI and bGNTII-D expression, their reaction products, and *N*-glycan. CBB staining, Western blotting analysis, and de-glycosylation analysis of cell lysate of (**a**) bGNTI- and (**b**) bGNTII-D-expressing Sf9 cells. Yellow and blue triangles indicate the *N*-glycosylated bGNTI and bGNTII-D, respectively. Gray triangles indicate the de-glycosylated bGNTI and bGNTII-D, respectively. *N*-Glycoproteins in the cell lysates were de-*N*-glycosylated by PNGase F digestion and detected by Western blotting using anti-His antibody. Triangles indicate the same as in Fig. 3A. (**c**) RP-HPLC analysis of the reaction products. bGNTI and bGNTII-D reactions were carried out using cell lysate, UDP-GlcNAc, and GN2M3 as a donor and an acceptor substrate, respectively. The elution position of the product was compared with authentic PA-sugar chains.

**Fig. 5:**
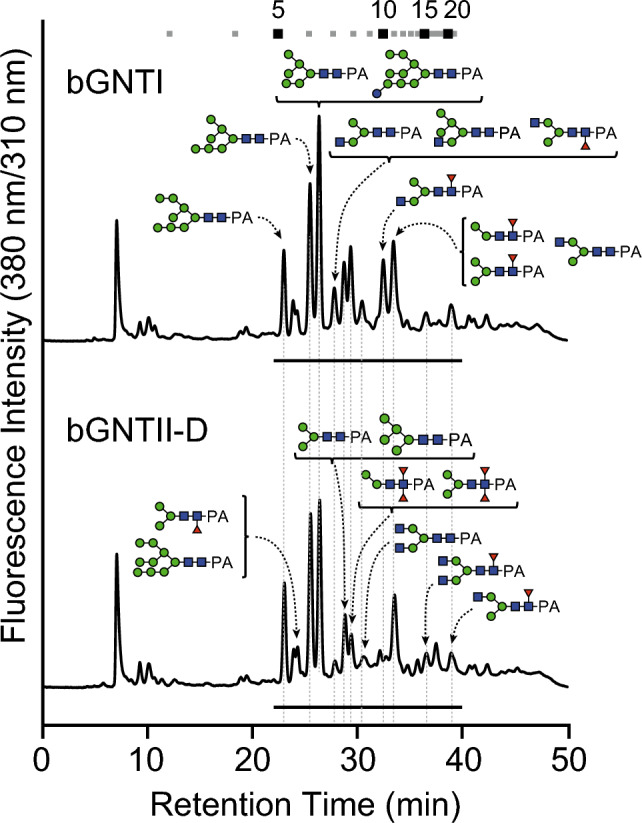
*N*-Glycan analysis of bGNTI- and bGNTII-D-expressing Sf9 cells. *N*-Glycans prepared from *N*-glycoproteins and labeled with PA were analyzed by RP-HPLC with a C18 column. The major structures are shown in chromatographs. Symbols indicate each sugar residue shown in Fig. 3.

The full-length bGNTI and bGNTII-D were expressed in Sf9 cells using BEVS and produced as *N*-glycoproteins (Fig. 4a and b). It should be noted that bGNTII has four splicing variants. Among them, bGNTII-D showed the highest in vitro activity^34^. When UDP-GlcNAc was used as the donor substrate, both purified recombinant proteins prepared from cell lysate exhibited GlcNAc transfer activities, resulting in the production of GNM5 and GN2M3, respectively (Fig. 4c). These results suggested that bGNTI and bGNTII-D may positively influence GN2M3 substrate biosynthesis. To clarify these possibilities, total *N*-glycan analysis was performed on Sf9 cells overexpressing bGNTI and bGNTII-D (Fig. 5 and Supplementary Table S2 online). *N*-Glycan analysis demonstrated successful remodeling of *N*-glycosylation pathway in bGNTI or bGNTII-D overexpression. Specifically, bGNTI overexpression led to the accumulation of *N*-glycans with terminal GlcNAc residues at the non-reducing terminal of Manα1,3-Man-R, such as GNM3FA, GNM5, and GNM5F. On the other hand, bGNTII-D overexpression led to the accumulation of *N*-glycans with terminal GlcNAc residues at the non-reducing terminal of Manα1,6-Man-R, such as derivatives of GNM3B and GN2M3. Compared to WT, the abundance of GNM3A-type *N*-glycan structures increased from 2.7 to 9.2%, while GNM3B- and GN2M3-type *N*-glycan structures increased from 0 to 13.9%, respectively. These results strongly indicated that the overexpression of bGNTI or bGNTII-D effectively contributed to the remodeling of the *N*-glycosylation pathway in insect cells.

**Fig. 6:**
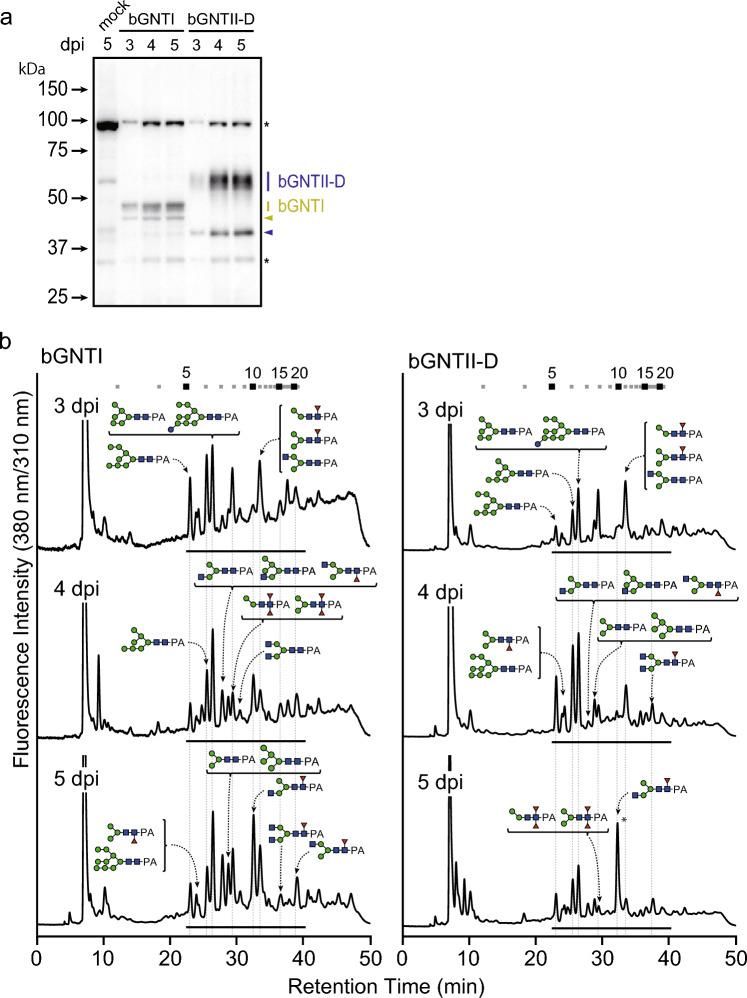
Temporal analyses of bGNTI and bGNTII-D expression and *N*-glycan changes. (**a**) Western blotting analysis of cell lysates prepared from 3 to 5 dpi Sf9 cells expressing bGNTI and bGNTII-D. Yellow and blue bars indicate the *N*-glycosylated bGNTI and bGNTII-D, respectively. Yellow and blue triangles indicate the non-*N*-glycosylated bGNTI and bGNTII-D, respectively. (**b**) *N*-Glycan analysis of bGNTI- or bGNTII-D-expressing Sf9 cells at 3–5 dpi. The peaks with asterisks contained PA-sugar chains and unknown impurities.

Generally, protein expression in BEVS enables the production of a larger amount of recombinant protein, but the production level and the time to reach maximum production levels depend on the target protein itself. Temporal analysis of bGNTI and bGNTII-D expression and the corresponding changes in *N*-glycan profiles were conducted to maximize the ratios of *N*-glycans with terminal GlcNAc residue(s) (Figs. 6, Supplementary Table S3 online). Both bGNTI and bGNTII-D were expressed from 3 days post-infection (dpi) and reached a plateau from 4 to 5 dpi. The overexpression of bGNTI and bGNTII-D resulted in an increase of the GNM3A-type *N*-glycan from 2.7% in the WT to 25.2% in Sf9 cells at 5 dpi by bGNTI expression and an increase of the GNM3B-type *N*-glycan from 0% in WT to 22.0% in Sf9 cells at 5 dpi due to bGNTII-D expression. In terms of the temporal change in the acceptor *N*-glycan for tri-antennary *N*-glycan biosynthesis, GN2M3-type *N*-glycan accumulated; however, the ratio was only 8.3%. These results suggest that the amounts of products were increased by the single expression of each *N*-acetylglucosaminyltransferase, but the effects were limited. It is also proposed that the simultaneous expression of multiple *N*-acetylglucosaminyltransferases and their combinations would be further required for the synthesis of tri-antennary *N*-glycan biosynthesis.

### Co-expression of *N*-acetylglucosaminyltransferases enhanced tri-antennary *N*-glycan biosynthesis

**Fig. 7:**
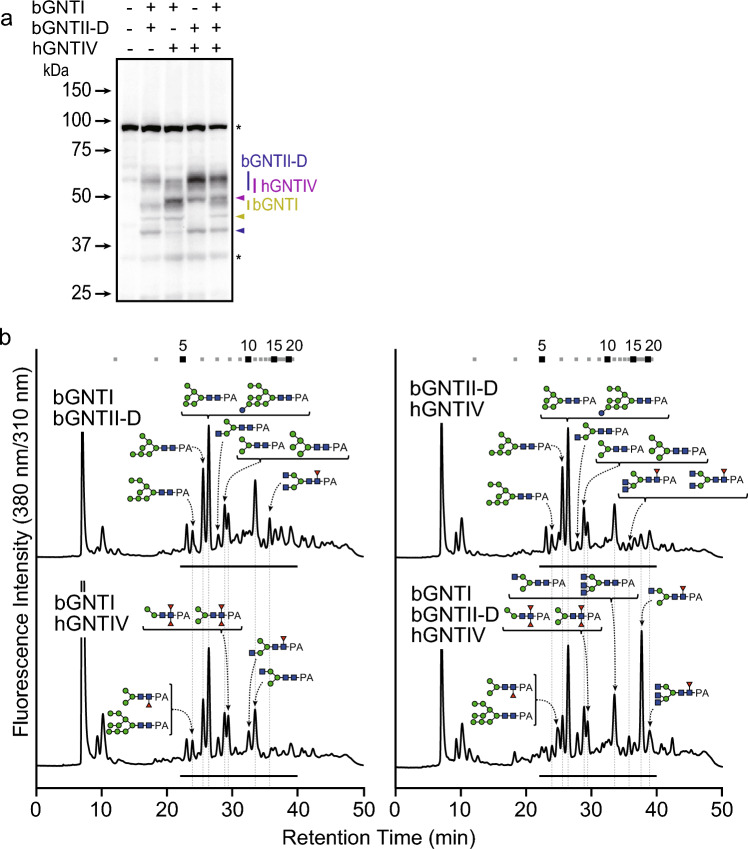
Analyses of expression of *N*-acetylglucosaminyltransferases and *N*-glycans in Sf9 cells expressing thems. (**a**) Western blotting analysis of bGNTI-, bGNTII-D-, and hGNTIV-expressing Sf9 cells. Yellow, blue, and purple triangles indicate non-*N*-glycosylated bGNTI-, bGNTII-D, and hGNTIV, respectively. (**b**) *N*-Glycan analysis of each Sf9 cell line expressing various combinations of *N*-acetylglucosaminyltransferases.

Expression of a single *N*-acetylglucosaminyltransferase alone was found to be insufficient for acceptor substrate accumulation and tri-antennary *N*-glycan biosynthesis. To overcome the difficulty of tri-antennary *N*-glycan biosynthesis, various combinations of *N*-acetylglucosaminyltransferase expression were examined, and the effects on the structural change of *N*-glycans were investigated. Each Sf9 cell line overexpressing *N*-acetylglucosaminyltransferases at 5 dpi was analyzed, as it was anticipated that the most pronounced structural changes in *N*-glycans would be observed, similar to those observed in cells overexpressing either bGNTI or bGNTII-D. Each cell line expressed *N*-acetylglucosaminyltransferases (Fig. 7a). The *N*-glycan structures in these cell lines were then determined (Fig. 7b, Supplementary Figure S7 and Table S4 online). The ratio of GN2M3-type *N*-glycan was the highest when bGNTI and bGNTII-D were co-expressed, reaching 12.8%. This was approximately double the ratio detected in the overexpression of bGNTII-D alone (6.4%). It is noteworthy that the ratio of GN3M3-type *N*-glycan was highest when bGNTI, bGNTII-D, and hGNTIV were co-expressed as expected, reaching 8.9%. Interestingly, the co-expression of bGNTI and hGNTIV had little effect on GN3M3-type *N*-glycan synthesis. However, while the effect was lower than that observed in the co-expression of bGNTI, bGNTII-D, and hGNTIV, the co-expression of bGNTII-D and hGNTIV resulted in the accumulation of GN3M3-type *N*-glycan. This suggested that bi-antennary *N*-glycan biosynthesis is a critical factor for tri-antennary *N*-glycan biosynthesis. These results indicated that the expression not only of GNTIV but also of other *N*-acetylglucosaminyltransferases, especially GNTII, was effective for the remodeling of the *N*-glycosylation pathway to biosynthesize tri-antennary *N*-glycan.

### Endogenous β-hexosaminidase negatively affected the accumulation of tri-antennary *N*-glycan

The presence of aberrant GN2M3-type *N*-glycans implies two possibilities: either the inhibition of GlcNAc transfer activity of bGNTII-D to the *N*-glycan due to the presence of β1,4-GlcNAc residue or the contribution of β-hexosaminidase. Co-expression of bGNTII-D and hGNTIV demonstrated an enhancement of tri-antennary *N*-glycan synthesis compared to the expression of GNTIV alone. Therefore, another possibility is that the contribution of β-hexosaminidase might be the main factor in producing the aberrant GN2M3-type *N*-glycans. A previous study identified three β-*N*-acetylglucosaminidases in Sf9 cells, SfGlcNAcase-1, SfGlcNAcase-3, and SfFDL^50–52^. Actually, the reaction products presumably produced by the action(s) of SfGlcNAcase-3 and SfFDL, specifically the GNM3B-type *N*-glycan, were also determined in all Sf9 cell lines.

**Fig. 8:**
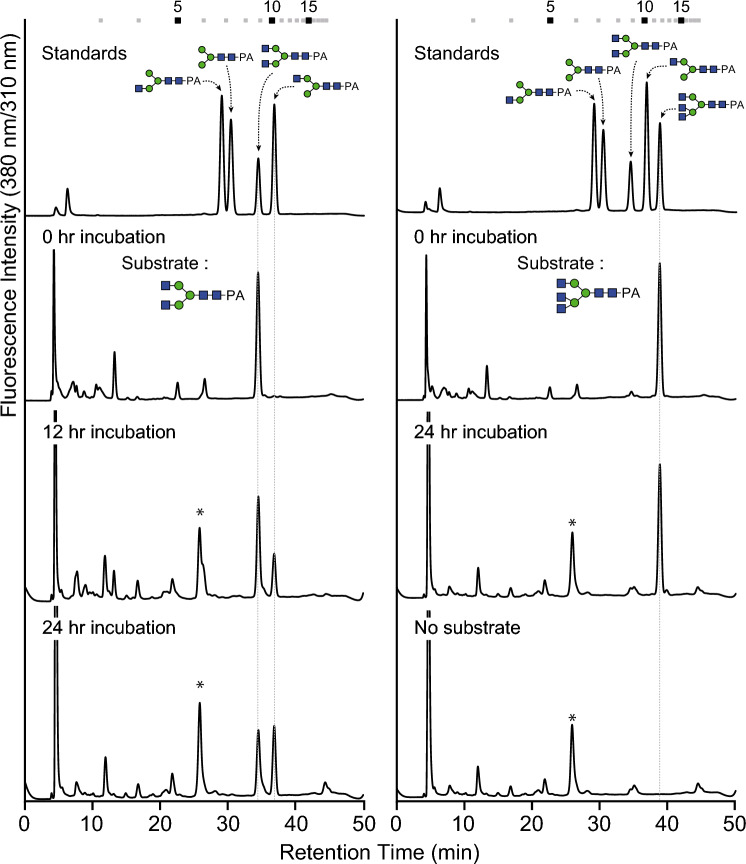
Detection of endogenous β-*N*-hexosaminidase activity toward GN2M3 and GN3M3. Each PA-sugar chain was reacted with cell lysate prepared from 3-day-old Sf9 cells after preculture. After the termination of the reactions, the supernatant was subjected to RP-HPLC and compared to the authentic PA-sugar chains. Asterisks indicate unknown peaks derived from the cell lysate.

To examine the endogenous β-*N*-acetylglucosaminidase activity, two PA-*N*-glycans, GN2M3-PA and GN3M3-PA, were incubated with cell lysate of Sf9 cells (Fig. 8). Analysis of the reaction products using GN2M3-PA was expected to result in three peaks corresponding to GNM3A, GNM3B, and M3. However, the cell lysate initially produced more GNM3B than GNM3A, indicating that SfGlcNAcase-3 and SfFDL preferentially degraded GN2M3 and predominantly generated GNM3B-type *N*-glycans due to their strong activities^51,52^. The scant presence of GNM3A also suggested the contribution of SfGlcNAcase-1, which has weaker activity and might therefore scarcely produce GNM3A. Interestingly, the cell lysate of Sf9 cells hardly produced the aberrant GN2M3-type *N*-glycan as a predominant product from GN3M3 (Fig. 8, right panel). This result indicated that, although β-*N*-acetylglucosaminidase(s) contributed to producing the pauci-mannosidic *N*-glycan from GN2M3, Sf9 cells exhibited weak hydrolytic activity toward GN3M3.

## Discussion

Insect cells could become a more attractive protein production platform by improving *N*-glycosylation, which is an important post-translational modification among various factors required for protein function^2^. Although there is a problem of virus contamination in insect cells^53–55^, it could be overcome by establishing and/or using virus-free insect cells^56^ or using *B. mori* with no reported virus contamination. In particular, *B. mori* is expected to be used as a next-generation “bioreactor” due to its stable and high protein productivity, the establishment of various genetic modification and genome-editing technologies^57–59^, and a baculovirus-based expression system using the silkworm as an expression host. This system enables the rapid and efficient production of target proteins^60^. Actually, some companies have tried to produce recombinant proteins, such as interferons for veterinary use, in *B. mori*^39,61^. However, protein expression by a *B. mori* transgenic gene expression system is time-consuming and laborious to obtain transformants. Additionally, a baculovirus-based expression system using the silkworm has the disadvantage of being difficult to evaluate, as changes and effects of post-translational modification are affected by endogenous factors present before virus infection. Therefore, an alternative system to evaluate insect post-translational modifications, especially *N*-glycosylation, and to apply it to *B. mori* transgenic gene expression system is desired. However, most research focusing on the modification of *N*-glycosylation has tried to establish the mammalian *N*-glycosylation pathway, particularly sialylation pathway^6,12^. Only a few studies in plants and CHO cells have focused on tri-antennary *N*-glycan synthesis^8,21–23^. Previous studies have demonstrated that some insect species had tiny amounts of tri-antennary *N*-glycan^25,26^, although an active insect GNTIV has not been identified^44^. It cannot be denied that this tri-antennary *N*-glycan might be a bi-antennary *N*-glycan with a GlcNAc or GalNAc residue. Indeed, insect *N*-acetylgalactosaminyltransferase (GalNAcT) has been identified and characterized^62,63^, and an insect GalNAcT has exhibited the synthetic activity of GlcNAcβ1,4-GlcNAcβ1,4-GlcNAc-R^32^. However, the monosaccharide composition of a tri-antennary *N*-glycan can, in principle, be identical to that of a bi-antennary structure bearing a terminal GalNAc residue; nevertheless, these structures can be distinguished based on RP-HPLC retention and/or MS/MS fragmentation patterns. Even though the presence of endogenous tri-antennary *N*-glycan is still controversial, there is no doubt that the ratio of abundance was quite low. Furthermore, some glycosyltransferase activities, such as GNTI and GNTII, are considered to be lower compared to mammalian cells^34,49^. Therefore, it is necessary to introduce not only the exogenous mammalian-derived GNTIV but also to overexpress or express additional GNTI and GNTII in order to modify the *N*-glycosylation pathway for effective tri-antennary *N*-glycan biosynthesis.

In this study, as a preliminary step toward the modification of *B. mori N*-glycosylation machinery, the effect of *N*-acetylglucosaminyltransferase expression on tri-antennary *N*-glycan biosynthesis in insect cells was investigated by exploring various combinations of expressions in Sf9 cells. From the standpoint of the localization of glycosyltransferases, the utilization of insect-derived GNTIV was preferable, because the introduction of exogenous glycosyltransferase sometimes leads to the production of aberrant *N*-glycan, presumably due to mis-localization in the Golgi^38^. Unfortunately, although we identified *B. mori* GNTIV, we could not determine its activity. Since the crystal structure of any GNTIV catalytic domain remains unclear, we could not speculate on which amino acid and/or domain in BmGNTIV is essential to exhibit GNTIV activity or how to modify BmGNTIV to show GNTIV activity. Then, hGNTIV was expressed, but no significant effect was confirmed by expression of GNTIV alone, and only 1.3% of tri-antennary *N*-glycans were synthesized. Kato et al. also reported that overexpression of GNTII enhanced bi-antennary *N*-glycan biosynthesis in *B. mori*^38^. Mabashi-Asazuma et al. demonstrated that co-expression of GNTII and β1,4-galactosyltransferase (GALT) enabled the production of bi-antennary galacto-*N*-glycan biosynthesis^35^. These results suggested that overexpression and co-expression of insect-derived *N*-acetylglucosaminyltransferase(s) and exogenous glycosyltransferase(s) would further improve bi-antennary *N*-glycan biosynthesis. Indeed, neither bGNTI nor bGNTII-D expression alone effectively accumulated bi-antennary *N*-glycans. However, the co-expression of bGNTI and bGNTII-D enhanced the accumulation of GN2M3-type *N*-glycans, which served as the acceptor substrate for GNTIV. Furthermore, co-expression of three *N*-acetylglucosaminyltransferases also revealed that not only GNTIV expression, but also both overexpression and co-expression of GNTI and GNTII were indispensable for the effective biosynthesis of tri-antennary *N*-glycans in insect cells.

It should be noted that the ratios of GNM3B-type *N*-glycan were also increased by bGNTII-D expression, indicating the possibility of further improvement in the ratios of GN2M3-type *N*-glycan. This is because, in general, GNM3B-type *N*-glycan is the hydrolysis product of GN2M3-type *N*-glycan by the action of hexosaminidase, especially FDL in insects. In fact, the crude cell lysate of Sf9 cells showed strong hydrolase activity of GlcNAc residue at the non-terminal residue of Manα1,3-Man-R and produced GNM3B from GN2M3 (Fig. 8, left panel). Therefore, it has been speculated that these hexosaminidase deficiencies are effective in the biosynthesis of bi-antennary *N*-glycan. In other words, the presence and the increased ratios of GNM3B-type *N*-glycan suggested that bGNTII-D was functional whereas hGNTIV was not functional, and that some *N*-glycoproteins were transported from the Golgi before being modified with β1,4-GlcNAc by hGNTIV. Once GN2M3-type *N*-glycan is further modified with β1,4-GlcNAc by hGNTIV, the resulting tri-antennary *N*-glycan exhibits greater tolerance against hexosaminidases compared to GN2M3-type *N*-glycan (Fig. 8, right panel). This leads to an increase in GN3M3-type *N*-glycans. However, the accumulation of GNM3B-type *N*-glycan was actually greater than that of GN3M3-type *N*-glycan. These results suggest that the correct localization of GNTIV is necessary for tri-antennary *N*-glycan biosynthesis. Taking into consideration the mammalian *N*-glycosylation pathway, GNTIV might act in *medial*- to *trans*-Golgi. The putative localization signal (transmembrane region of GNTIV, Val7-Leu21) triggered mis-localization in insect cells, as mentioned previously^38^. Interestingly, *N*-glycan analysis of Sf9 cells expressing *N*-acetylglucosaminyltransferase(s) demonstrated a drastic reduction in fucosylation of *N*-glycans, especially M3F, compared to WT, to half to one- third of the level (Supplementary Tables S1, S2, and S4). Although the substrate specificities of insect fucosyltransferases (FUTs) toward tri-antennary *N*-glycan are unknown, this observation led to the speculation that hGNTIV competes with α1,3- and/or α1,6-fucosyltransferases for the acceptor substrate of GN2M3^64^. Thus, if this competitive reaction with FUTs could be avoided, a more effective biosynthesis of tri-antennary *N*-glycan would be possible. In the case of plants, the utilization and replacement of the regions of cytosolic and transmembrane of glycosyltransferases has been shown to be effective for the correct localization of exogenous glycosyltransferases and their function^65–69^. This is also the case for insect cells, demonstrating that replacement of this region of cytosolic and transmembrane is effective^37^. Therefore, the utilization of the cytosolic and transmembrane regions of endogenous *N*-glycosylation enzymes is considered to be more preferable for the efficient modification of the *N*-glycosylation pathway using exogenous *N*-glycosylation enzymes. The candidates of the regions are those of insect *N*-glycosylation enzymes that probably function in the *medial*-Golgi, such as GNTII, mannosidase II, and FUTs. A chimeric enzyme consisting of a localization signal from insects and a catalytic domain of glycosyltransferase would provide a mammalian-like *N*-glycosylation system and a useful protein production platform in insects.

In this study, we demonstrated the modification of insect *N*-glycosylation for the biosynthesis of mammalian-type *N*-glycans. However, as demonstrated in *N*-glycan analysis of WT Sf9 cells, insect *N*-glycans are generally considered to consist mostly of oligomannosidic structures and/or pauci-mannose types with core fucose residue(s)^24,47,70^. Thus, inhibition of the biosynthesis of these *N*-glycans is indispensable for mammalian-type *N*-glycan biosynthesis. The well-established and studied approach involves the inactivation, down-regulation, or knockout of *N*-acetylhexosaminidases^52,71–73^. However, the localizations of *N*-acetylhexosaminidases have been controversial^51,74^, but GlcNAc digestion by *N*-acetylhexosaminidases in insect cells is considered to occur as a post-Golgi modification^52^. Therefore, the strategy for the inactivation of *N*-acetylhexosaminidases is sufficient for the production of bi-antennary *N*-glycan GN2M3 but insufficient or not suitable for bi-antennary and more branched mammalian-type *N*-glycan biosynthesis. This evidence suggests the importance of *N*-glycosylation in the Golgi: the mammalization of *N*-glycan should be completed in the Golgi. Therefore, both the accumulation of GN2M3, which is used as an acceptor substrate for various mammalian-derived glycosyltransferases, and the efficient function of glycosyltransferases must be simultaneously ensured. Interestingly, *B. mori* MSG accumulates GN2M3 as a predominant *N*-glycan^43^. This is presumably due to the low expression levels and/or activities of *N*-acetylhexosaminidases, particularly FDL. This indicates that overexpressing *N*-acetylglucosaminyltransferases in MSG could potentially synthesize mammalian-type *N*-glycans the most efficiently. In other words, the *N*-glycan structures change in a tissue-, growth-, and glycosylation-related enzyme expression-dependent manner. Therefore, it is critical to determine the location and type of tissues to target for modifying of *N*-glycan structures to achieve the conversion of insect-type into mammalian-type *N*-glycans in the case of actual insects. In future work, it will be necessary to demonstrate that mammalian-type *N*-glycan biosynthesis could be effectively achieved by applying the results obtained in this study and verifying its effectiveness not only in other cultured cells but also in actual insects such as the silkworm *B. mori*.

## Materials and methods

### Materials

UDP-GlcNAc was purchased from YAMASA (Chiba, Japan). 2-Pyridylaminated (PA)-sugar chains were purchased from TaKaRa Bio (Shiga, Japan) and Masuda Chemical Industry (Kagawa, Japan) and prepared as previously reported^33^. The PA-sugar chains used in this study are listed in Supplementary Fig. S1 online.

### Insect cells

*Spodoptera frugiperda* Sf9 cells were maintained at 25 °C in Sf-900 III SFM (Gibco, Eggenstein, Germany) containing 10% fetal calf serum (Gibco).

### Expression of *N*-acetylglucosaminyltransferases in Sf9 cells and purification of BmGNTIV

The codon-optimized cDNA corresponding to the full-length *B. mori* GNTIV homologue for the expression in Sf9 cells, with a hexa-histidine tag at C-terminus, was prepared by PCR using KOD plus Neo polymerase (TOYOBO, Osaka, Japan). The synthesized cDNA (Invitrogen, Carlsbad, CA) served as a template, alone with the primer set listed in Supplementary Table S5 online. To produce BmGNTIV as a soluble and secreted protein, a truncated form of BmGNTIV without a putative cytoplasmic and transmembrane region was amplified using KOD plus Neo polymerase. Full-length cDNA served as a template, alone with primer set listed in Supplementary Table S5 online. This truncated form was then introduced into the pFastBac vector (Invitrogen) with a GP67 signal at the 5’-upstream region, as previously reported^32,33^. cDNA fragments encoding full-length BmGNTI and BmGNTII were prepared from the template used in previous studies^34^, alone with the primer sets listed in Supplementary Table S5 online. The full-length *N*-acetylglucosaminyltransferases were ligated with pFastBac™ vector (Invitrogen). The open reading frame of human GNTIVa (hGNTIV) was obtained from Horizon Discovery (Cambridge, UK) and the corresponding expression vector for use in Sf9 cells was constructed as described above.

Baculovirus for the production of each *N*-acetylglucosaminyltransferase was prepared using the Bac-to-Bac expression system and Cellfectin® II Reagent Transfection Reagent (Invtrogen). After amplification of the P2 baculovirus, a soluble and secreted form of BmGNTIV was expressed in Sf9 cells as previously reported^32,33^. In brief, a total of 1.0 × 10^8^ Sf9 cells in 100 ml medium were transfected with P2 baculovirus and cultivated at 25 °C, 120 rpm, for 4 days. In the case of co-expression, the titers of P2 baculoviruses were measured and the same titers of baculoviruses were used. The medium was collected and centrifuged at 4 °C, 1000×*g* for 5 min. To precipitate proteins, ammonium sulfate precipitation was performed. The precipitant was dissolved in 50 mM Tris–HCl buffer, pH 7.5, 500 mM NaCl, and 5 mM imidazole (buffer A), followed by dialysis against buffer A overnight. The solution was subjected to a TALON® Metal Affinity Resin column (TaKaRa, Shiga, Japan) equilibrated with buffer A. After washing with a 10-times-column volume of buffer A, the recombinant enzyme was eluted with buffer A containing 200 mM imidazole. The eluate was dialyzed against 50 mM Tris–HCl pH 7.5, 300 mM NaCl (buffer B) for 3 h, followed by measurement of the protein concentration and the addition of glycerol and buffer B to a final concentration of 20 mM Tris–HCl pH 7.5, 150 mM NaCl, 50% glycerol. The enzyme solution was stored at –20 °C.

### Protein analysis

Each group of *N*-acetylglucosaminyltransferase-expressing Sf9 cells was collected by centrifugation at 4 °C, 1500×*g*, for 10 min. The cells were washed once with PBS buffer, solubilized in lysis buffer (PBS, 1% Triton X-100), and kept on ice for 1 h. The solution was further sonicated for 10 s and then centrifuged at 4 °C and 10,000×g for 10 min. The cell lysate was used for protein analysis.

The cell lysates were separated by 5–20% SDS-PAGE and detected by CBB staining or Western blotting. In Western blotting analysis, anti-His-tag antibody (FUJIFILM Wako Pure Chemical, Osaka, Japan) and anti-mouse antibody conjugated to horseradish peroxidase (GE Healthcare, Tokyo, Japan) were used as the primary and secondary antibodies, respectively. In hGNTIV detection, anti-GnTIV-A mouse antibody (Sigma-Aldrich, St. Louis, MO) and anti-mouse antibody conjugated to horseradish peroxidase (GE Healthcare) were used as the primary and secondary antibodies, respectively. The specific signals were visualized using Luminate Forte western HRP Substrate (Millipore, Billerica, MA) and an iBright Imaging System (Invitrogen).

The cell lysates of baculovirus-infected Sf9 cells were digested with Peptide:*N*-glycosidase F (PNGase F, TaKaRa) following the manufacturer’s protocols, followed by separation by 5–20% SDS-PAGE and visualization by Western blotting using suitable anti-bodies for each protein.

### Activity assays

BmGNTIV and hGNTIV activities were examined in a total volume of 50 μl containing 50 mM sodium cacodylate, using 50 ng of purified protein of BmGNTIV or crude cell lysate of hGNTIV-expressing Sf9 cells. The reaction mixture also contained 40 mM UDP-GlcNAc and 100 nM GN2M3 as donor and acceptor substrates, respectively. The mixture was then incubated at 37 °C for 12 h in the presence of 15 mM MnCl_2_. The optimal buffer, temperatures, pH, and ion dependence were determined using methods previously reported^32,33^. BmGNTI and BmGNTII activities were determined as previously reported^34^.

The reactions were terminated by incubation at 100 °C for 5 min, and the reaction products were centrifuged at 4 °C, 10,000×*g* for 5 min. The supernatant was analyzed using high-performance liquid chromatography (HPLC) as described later. The eluate obtained from the HPLC analysis, which contained the reaction product, was corrected and further subjected to LC–MS/MS using an Eksigent M5 MicroLC system (SCIEX, Marlborough, MA) coupled to a TripleTOF™ 6600 mass spectrometer (SCIEX). For LC separation, the mobile phase consisted of 0.01% formic acid in water (solvent A) and water/acetic acid containing 0.01% formic acid (solvent B, 80/20, v/v). The reaction product was separated on a Kinetex® 2.6 μm XB-C18 100 Å column (0.3 mm i.d. × 150 mm; Phenomenex, Torrance, CA) using a linear gradient from 5 to 50% solvent B over 20 min at a flow rate of 5 μL/min. OptiFlow was used as the ionization source. Ion source gas parameters were set as follows: GS1 25, GS2 50, and Curtain Gas 20. The ion spray voltage was set to 4500 V, and the source temperature was maintained at 75 °C. MS/MS spectra were acquired using a targeted acquisition method, in which specific precursor ions (*m/z* 799.8) were selected for fragmentation. The mass range was set to *m/z* 100–1000 for Experiment 1 (MS scan) and *m/z* 150–2000 for Experiment 2 (MS/MS scan). The accumulation times were 200 ms and 150 ms for Experiment 1 and Experiment 2, respectively. Up to top three precursor ions were selected per cycle for MS/MS, and the collision energy and collision energy spread for Experiment 2 were set to 45 and 15, respectively. Data was analyzed using SCIEX OS version 3.1.5.3945 (SCIEX).

### Determination of neutral *N*-glycans

Baculovirus-transfected Sf9 cells were corrected by centrifugation at 4 °C, 1500×*g* for 5 min, and then washed with PBS five times. The corrected cells were lysed with a lysis buffer (PBS, 1% Triton X-100) on ice for 1 h. The lysed product was then centrifuged at 4 °C, 15,000×*g* for 5 min. Crude proteins in the supernatant were precipitated by acetone and centrifuged at 4 °C, 15,000×*g* for 20 min. The pellet was further lyophilized and used for *N*-glycan preparation.

Hydrazinolysis of *N*-glycoproteins and PA-labeling of the neutral *N*-glycans were performed as previously described^33^. The excess amount of the unreacted PA was removed by size-fractionation (SF)-HPLC using a Shodex Asahipak NH2P-50 4E column (4.6 × 250 mm, Showa Denko Co., Ltd., Tokyo, Japan) with the Hitachi LaChrom system (Hitachi High-Technologies, Tokyo, Japan). The eluted fractions were monitored by measuring fluorescence intensity using excitation and emission wavelengths of 310 and 380 nm, respectively. The purified PA-sugar chains were separated by reverse-phase (RP)-HPLC and collected. The PA-sugar chains were further analyzed by liquid-chromatography tandem mass spectrometry (LC–MS/MS) using an Agilent Technologies 1200 series instrument (Agilent Technologies, Santa Clara, CA) equipped with amazon ETD (Bruker Daltonics, Bremen, Germany). LC–MS/MS analysis was performed under conditions previously described^33^. The MS data were analyzed using DataAnalysis 4.0 software (Bruker Daltonics).

### HPLC analyses

The analysis of PA-sugar chains from the reaction product and *N*-glycoproteins involved RP-HPLC or SF-HPLC. In RP-HPLC analysis, the mobile phases were composed of water/acetonitrile containing 0.02% trifluoroacetic acid (solvent A: 100/0 v/v; solvent B: 80/20 v/v). An analytical column of Cosmosil C18 column (4.6 × 250 mm, nacalai tesque, Kyoto, Japan) was employed, with elution achieved by linearly increasing the solvent B concentration from 0 to 25% over 35 min at a flow rate of 0.7 ml/min. In SF-HPLC, the mobile phases were composed of water/acetonitrile/acetic acid/triethylamine (solvent C: 0/98/2/0 v/v; solvent D: 92/0/5/3 v/v). An analytical column of Asahipak NH2P-50 2D, 2.0 mmID × 150 mm (Showa Denko Co., Ltd.) was utilized, with PA-sugar chains eluted by linearly increasing the solvent D concentration from 20 to 55% over 35 min at a flow rate of 0.2 ml/min. All of the eluates were monitored by measuring the fluorescence intensity using excitation and emission wavelengths of 310 and 380 nm, respectively^75^.

### Detection of endogenous hexosaminidase activities

On the third day post-inoculation, Sf9 cells were harvested by centrifugation at 4 °C, 1500×*g* for 5 min and subsequently washed five times with PBS. The collected cells were lysed using a lysis buffer containing 1% Triton X-100 in 50 mM acetic acid pH 5.0 on ice for 1 h. Following lysis, the cellular debris was removed by centrifugation at 4 °C, 15,000×*g* for 5 min, and the protein concentration of the resultant supernatant was determined using the Bradford assay with Protein assay CBB solution(5x) (nacalai tesque). Ten micrograms of total protein from cell lysate was incubated with GN2M3 or GN3M3 for approximately 16 h at 27 °C. The reaction was halted by incubation at 100 °C for 5 min, followed by centrifugation at 4 °C, 10,000×*g* for 5 to recover the supernatant, which was then analyzed by RP-HPLC.

## Supplementary Information

Supplementary Information.

## Data Availability

The datasets used and/or analyzed during the current study are available from the corresponding author on reasonable request. The gene sequences used in this study, *BmGNTI* , *BmGNTII* , *BmGNTIV* , and *hGNTIV* , were obtained from the databases of KEGG, SGID, and GenBank, with accession numbers KEGG Entry No. 100653407, SGID Entry KWMTBOMO06300 and KWMTBOMO00036, and NM\_012214.3.
