# Supplementary material for: N-Glycoengineering of insect cells for tri-antennary N-glycan biosynthesis
Source: Sci Rep. 2026 Mar 16;16:15012. doi: 10.1038/s41598-026-41152-8 (PMC13172561; doi:10.1038/s41598-026-41152-8)
Supplement: Supplementary file 1 — Supplementary Information. [file 41598_2026_41152_MOESM1_ESM.pdf]

N-Glycoengineering of insect for tri-antennary *N*-glycan biosynthesis

Hiroyuki Kajiura[1,2,†], Naokuni Nishiguchi[1,3,†], Reimi Lai Sang Sawada-Choi[1,3], Yuto Sana[1,3], Ryo Misaki[1,2] and Kazuhito Fujiyama[1,2,4*]

[1]International Center for Biotechnology, Osaka University, 2-1 Yamada-oka, Suita, Osaka 565-0871, Japan

[2]Institute for Open and Transdisciplinary Research Initiatives (OTRI), Osaka University, 2-1 Yamada-oka, Suita-shi, Osaka 565-0871, Japan

[3]Department of Biotechnology, Graduate School of Engineering, Osaka University, 2-1 Yamada-oka, Suita, Osaka 565-0871, Japan

[4]Osaka University Cooperative Research Station in Southeast Asia (OU:CRS), Faculty of Science, Mahidol University, Bangkok, Thailand

†: These authors equally contributed to this work.

*Correspondence: Kazuhito Fujiyama, fujiyama@icb.osaka-u.ac.jp (K. Fujiyama).

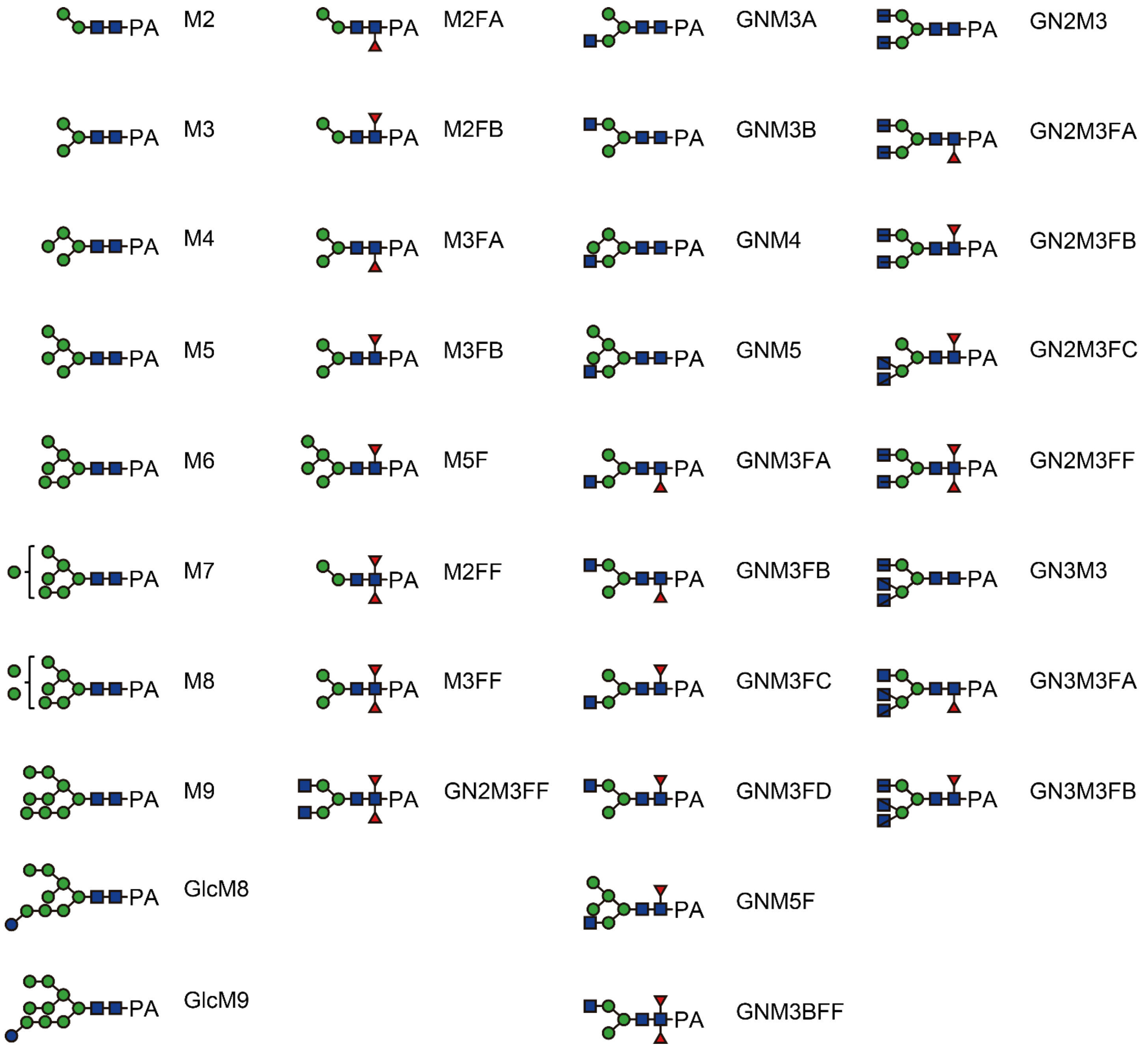

Supplementary Fig. S1 Structures of the PA-sugar chains used in this study and their abbreviations.

Symbols are the same as shown in Figure 2

```
KEGG    MIDIRRRLKYLVCAVVRICGGWCLNQRIRLCVFVMSLMCAFFTILVLASASYYVCKRSLR
SGID    ------------------------------------------------------------

KEGG    ECAATLGVHSPPDYSPSQRIVHAHSAQNESMAKKITSLEEKLNNFEVRYRVKPVTYNFPK
SGID    ------------------------------MAKKITSLEEKLNNFEVRYRVKPVTYNFPK
                                      ******************************

KEGG    ETAPYPPGYSEEAMNMIHNLTGTRVLNGLQPRYIHRMQLPFLVQILTHLLNDPFSLWPLY
SGID    ETAPYPPGYSEEAMNMIHNLTGTRVLNGLQPRYIHRMQLPFLVQILTHLLNDPFSLWPLY
        ************************************************************

KEGG    HLSGARHYMDFVIGIPTVKRDKQTYLFTTLTYLIKALSAEDKRSVVIVILVGEPDVTYIL
SGID    HLSGARHYMDFVIGIPTVKRDKQTYLFTTLTYLIKALSAEDKRSVVIVILVGEPDVTYIL
        ************************************************************

KEGG    KLARQIEIMFPREISDGVIEILAPSPAYYPELEDLCPTLGDSPKRAMWRTKQNLDNIYLM
SGID    KLARQIEIMFPREISDGVIEILAPSPAYYPELEDLCPTLGDSPKRAMWRTKQNLDNIYLM
        ************************************************************

KEGG    AYAISKGVYYLMLEDDVTTKKDFLPEMKGYIKTTTEKTPHWIFIEFCQVGAIGKVFRTRD
SGID    AYAISKGVYYLMLEDDVTTKKDFLPEMKGYIKTTTEKTPHWIFIEFCQVGAIGKVFRTRD
        ************************************************************

KEGG    LLPFVTYSQIFYSNMPIDWLLESYLADRVCSIDKKSLCPAAAKKYKRLEKKSCAESKLRV
SGID    LLPFVTYSQIFYSNMPIDWLLESYLADRVCSIDKK--------------SKSCAESKLRV
        ***********************************              .**********

KEGG    RPRYKVSLFQHIGVYSSLQGKIQKVQDPQFGKVQSYFPHQNPPAQKITTTIEDYYQHSIQ
SGID    RPRYKVSLFQHIGVYSSLQGKIQKVQDPQFGKVQSYFPHQNPPAQKITTTIEDYYQHSIQ
        ************************************************************

KEGG    NAYEGIDFFWGKKPKKGDTLEFWYGRPLQIKRVTFRSGNAEHITDQFYNTVVEVLPAFGD
SGID    NAYEGIDFFWGKKPKKGDTLEFWYGRPLQIKRVTFRSGNAEHITDQFYNTVVEVLPAFGD
        ************************************************************

KEGG    NNFTTILHFDEFGLADGDVEEEFSLVKAIRLRVNADSKYWVILSEIYIQTPDEKKT----
SGID    NNFTTILHFDEFGLADGDVEEEFSLVKAIRLRVNADSKYWVILSEEYAIFHTYREYREEV
        ********************************************* *      ::

KEGG    --------
SGID    HNANIFFK
```

Supplementary Fig. S2 Alignment of the amino acid sequences of GNTIVs.

Amino acid sequences of putative BmGNTIVs deposited in KEGG or the silkworm genome informative database (SGID) were aligned using CLUSTALW (http://align.genome.jp/). Identical, similar, and semi-conservative replacements of amino acids are indicated by an asterisk, colon, and dot, respectively.

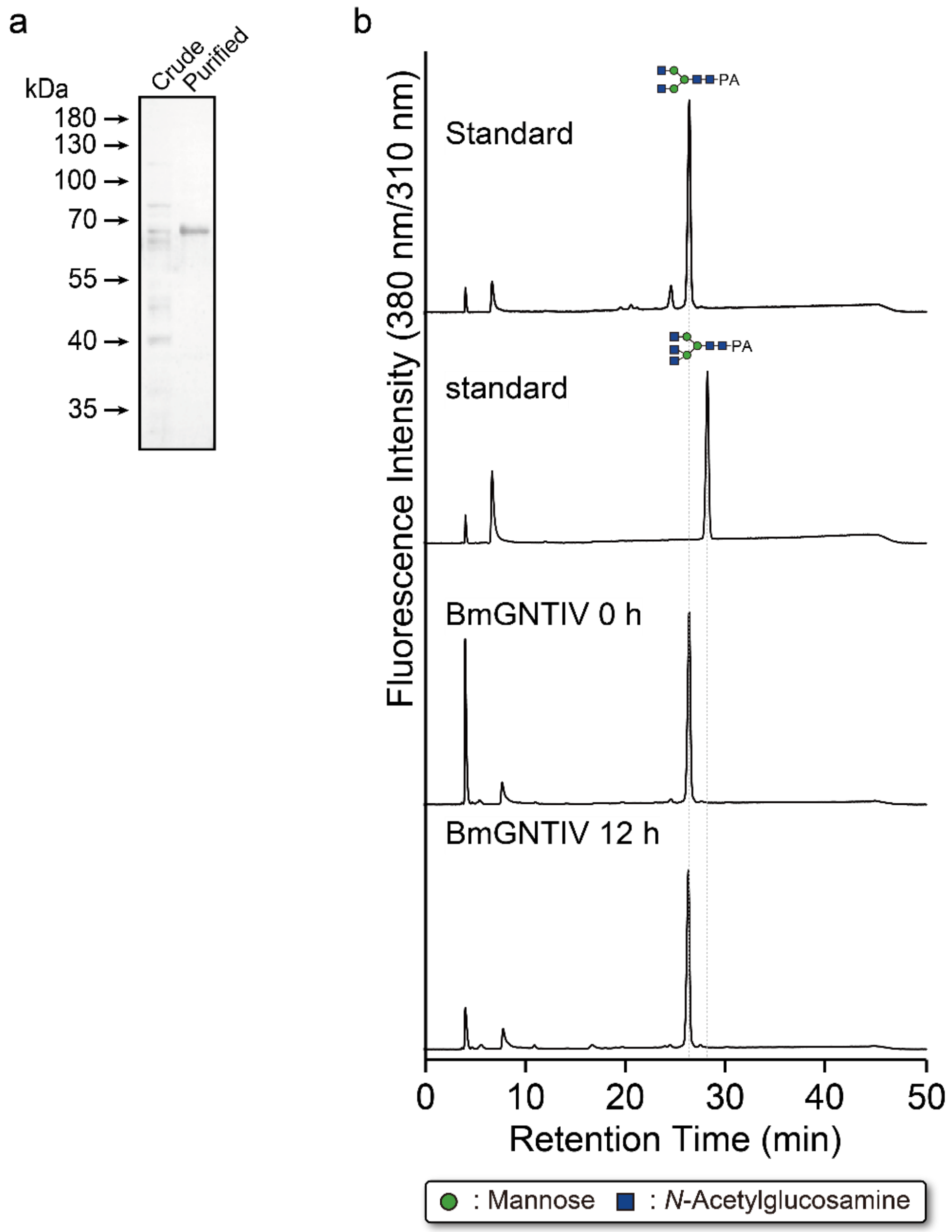

Supplementary Fig. S3 Expression and purification of BmGNTIV, which lacked detectable activity.

(a) Western blotting analysis of cell lysate of BmGNTIV-expressing Sf9 cells and purified BmGNTIV.

(b) RP-HPLC analysis of the reaction products. BmGNTIV reaction was carried out using purified BmGNTIV, UDP-GlcNAc, and GN2M3 as a donor and an acceptor substrate, respectively. The elution position of the product was compared with authentic PA-sugar chains.

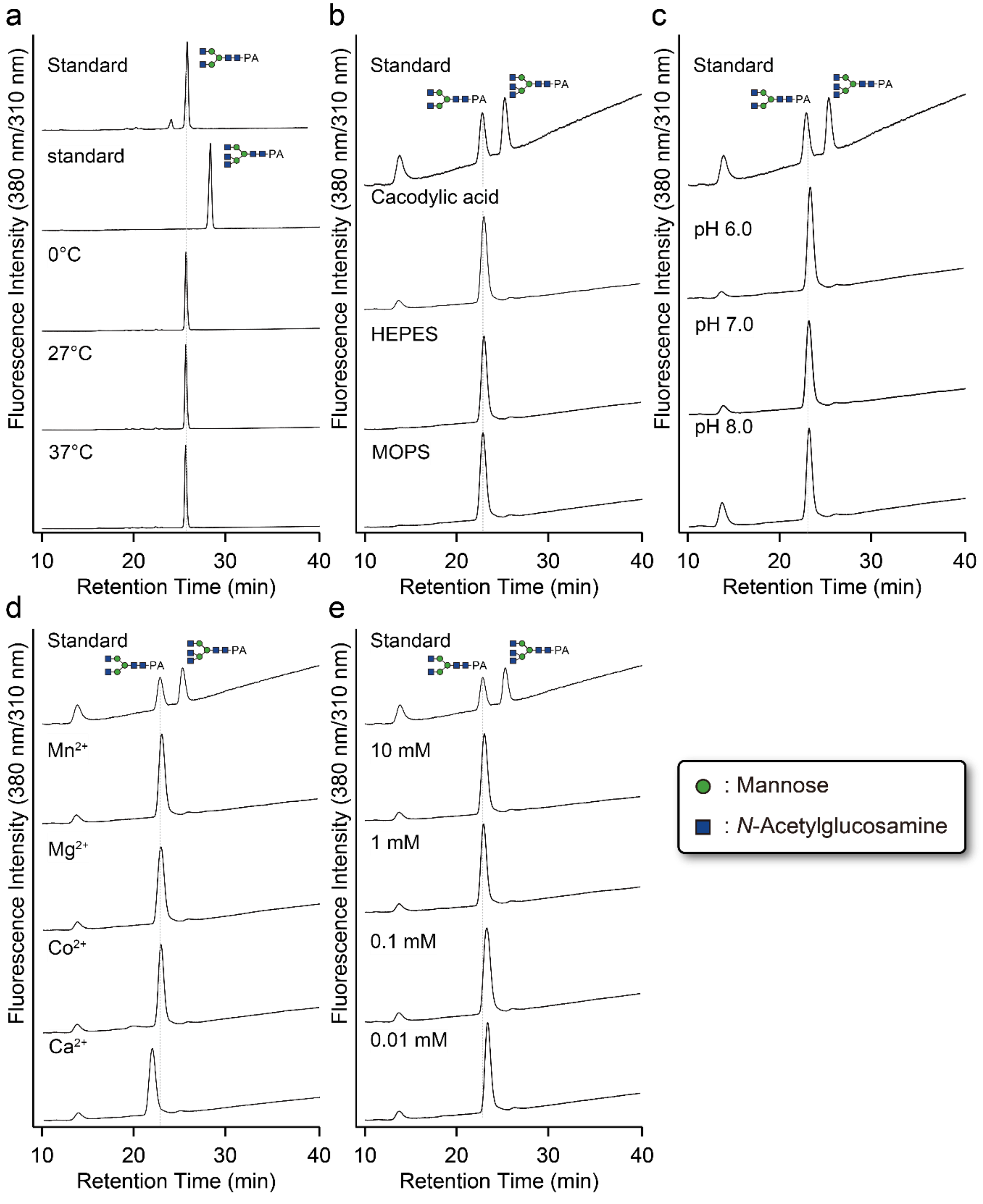

Supplementary Fig. S4 Activity assay of recombinant BmGNTIV.

Activities under the various conditions of BmGNTIV, such as (a) temperature, (b) buffers, (c) pHs, (d) metal ions, or (e) concentrations of Mn^{2+}, were examined.

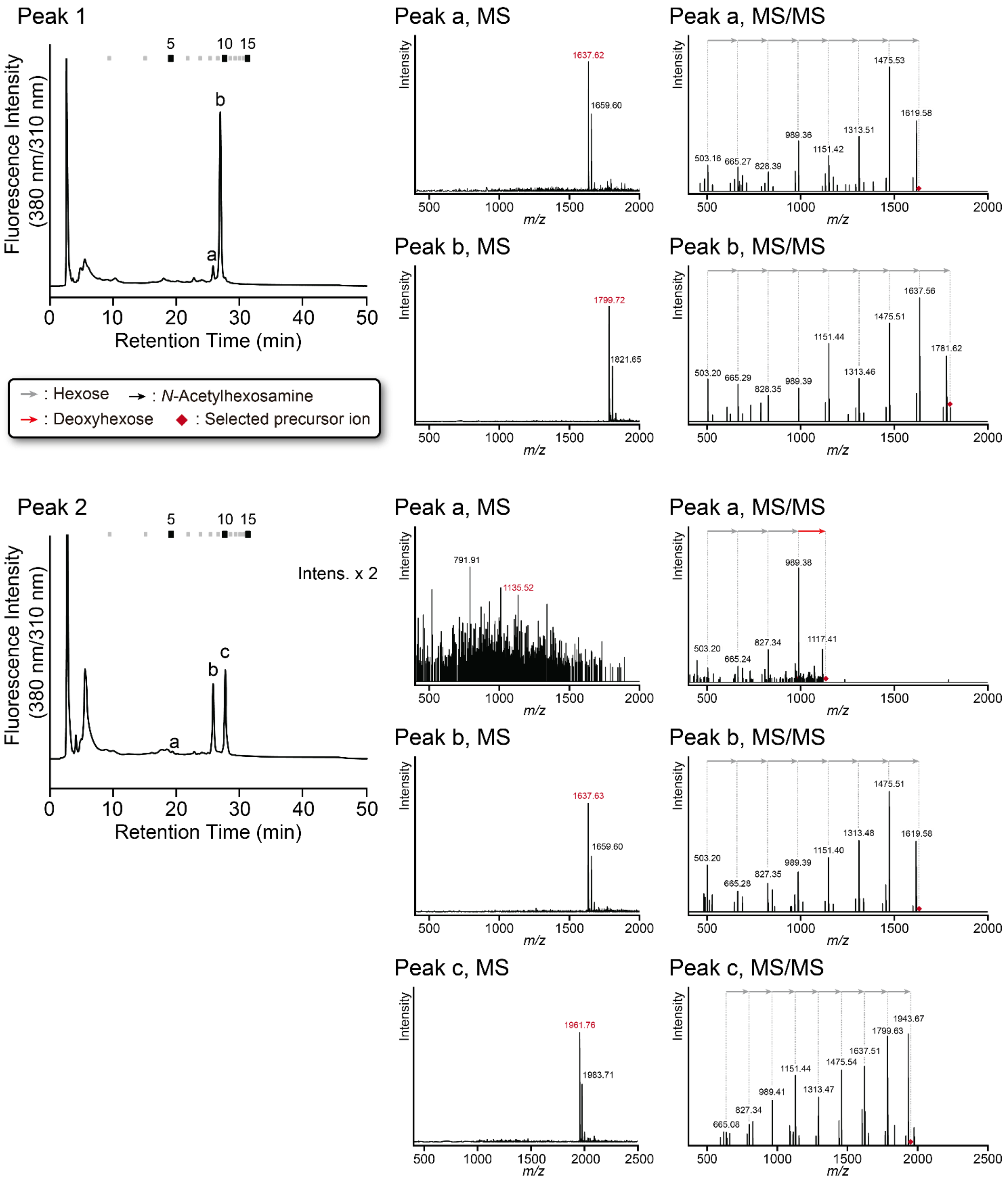

Supplementary Fig. S5 Structural analysis of *N*-glycans derived from WT by LC-MS/MS.

PA-labeled *N*-glycans collected by RP-HPLC were further analyzed by LC-MS/MS following size-fractionation HPLC using an ion trap mass spectrometer. Red numbers in the MS spectra indicate precursor ions selected for subsequent MS/MS analysis. In the MS/MS spectra, gray, black, and red arrows denote hexose, *N*-acetylhexosamine, and deoxyhexose residues, respectively. Red diamonds indicate the molecular weights of the precursor ions. Asterisks in the HPLC chromatograms indicate peaks whose molecular weights, as determined by mass spectrometry, did not correspond to those of PA-labeled *N*-glycans.

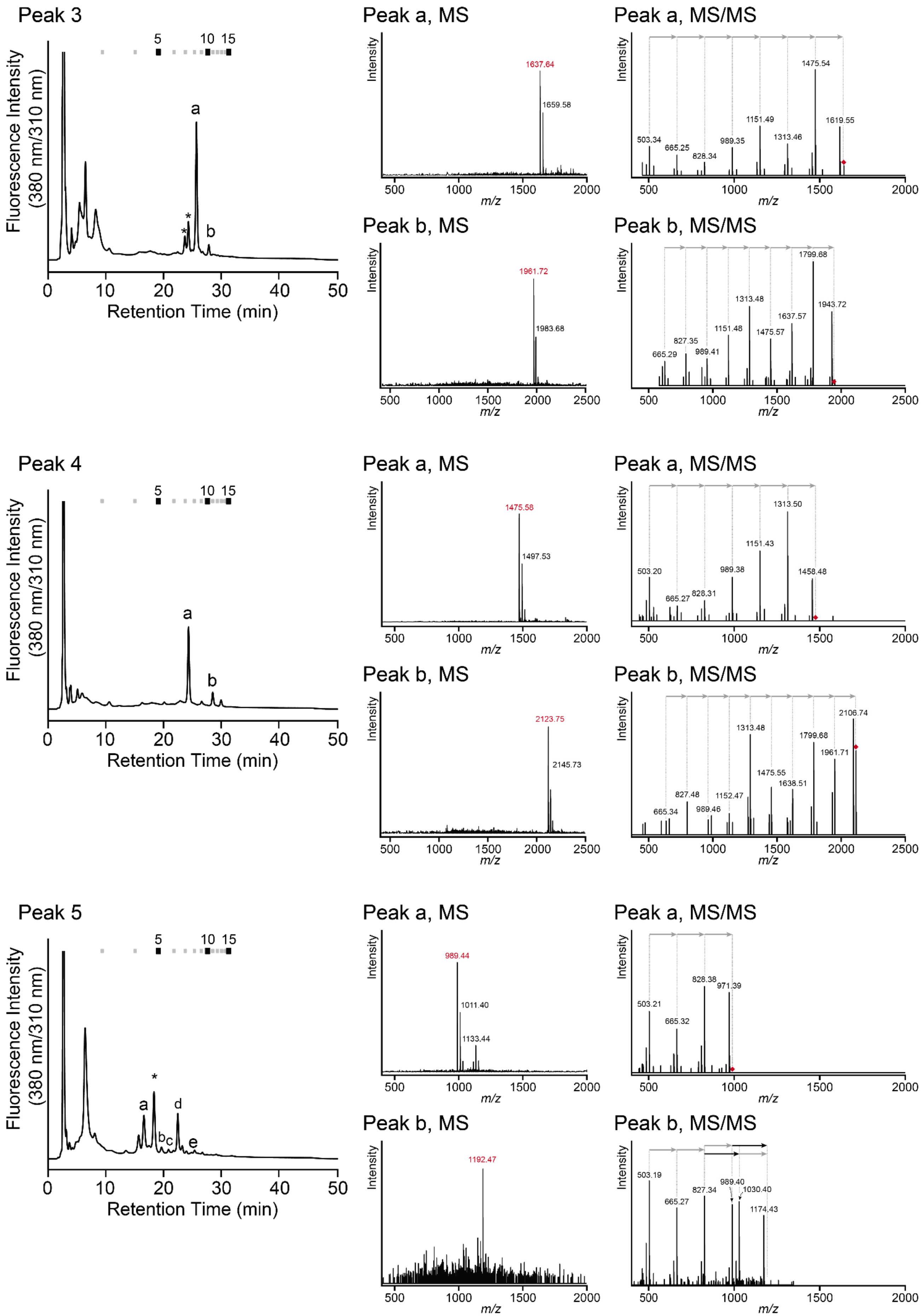

Supplementary Fig. S5 (continued)

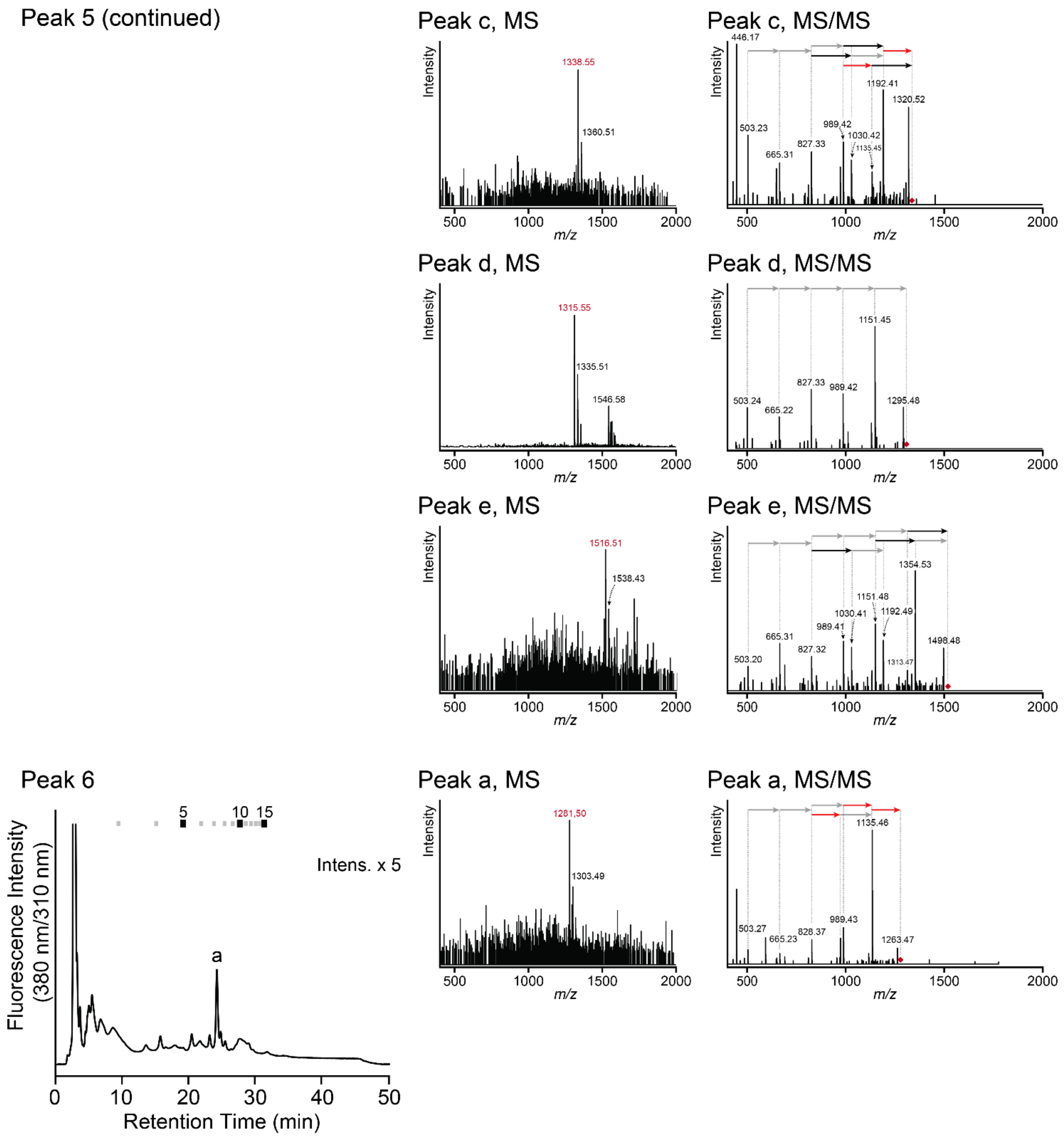

Supplementary Fig. S5 (continued)

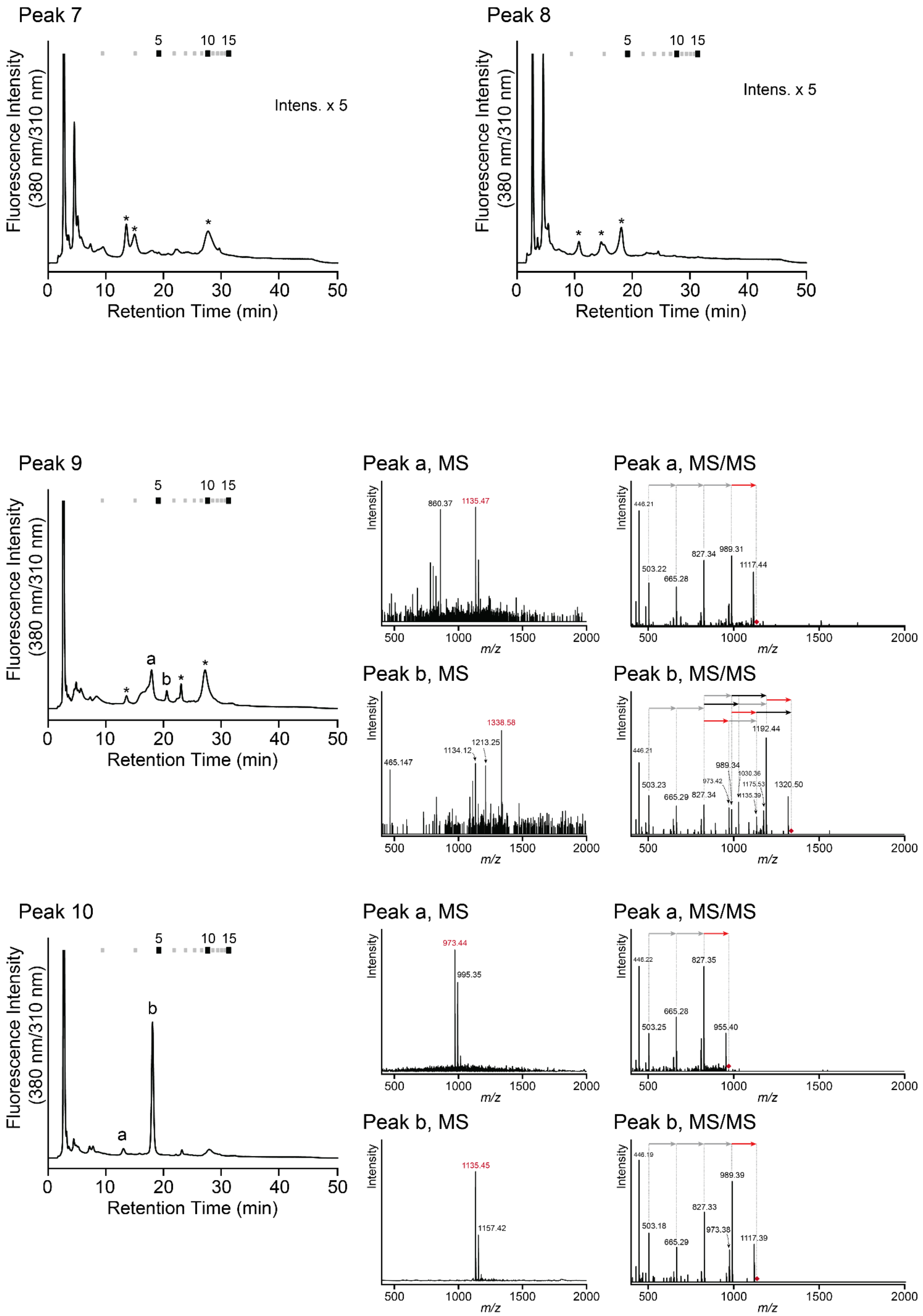

Supplementary Fig. S5 (continued)

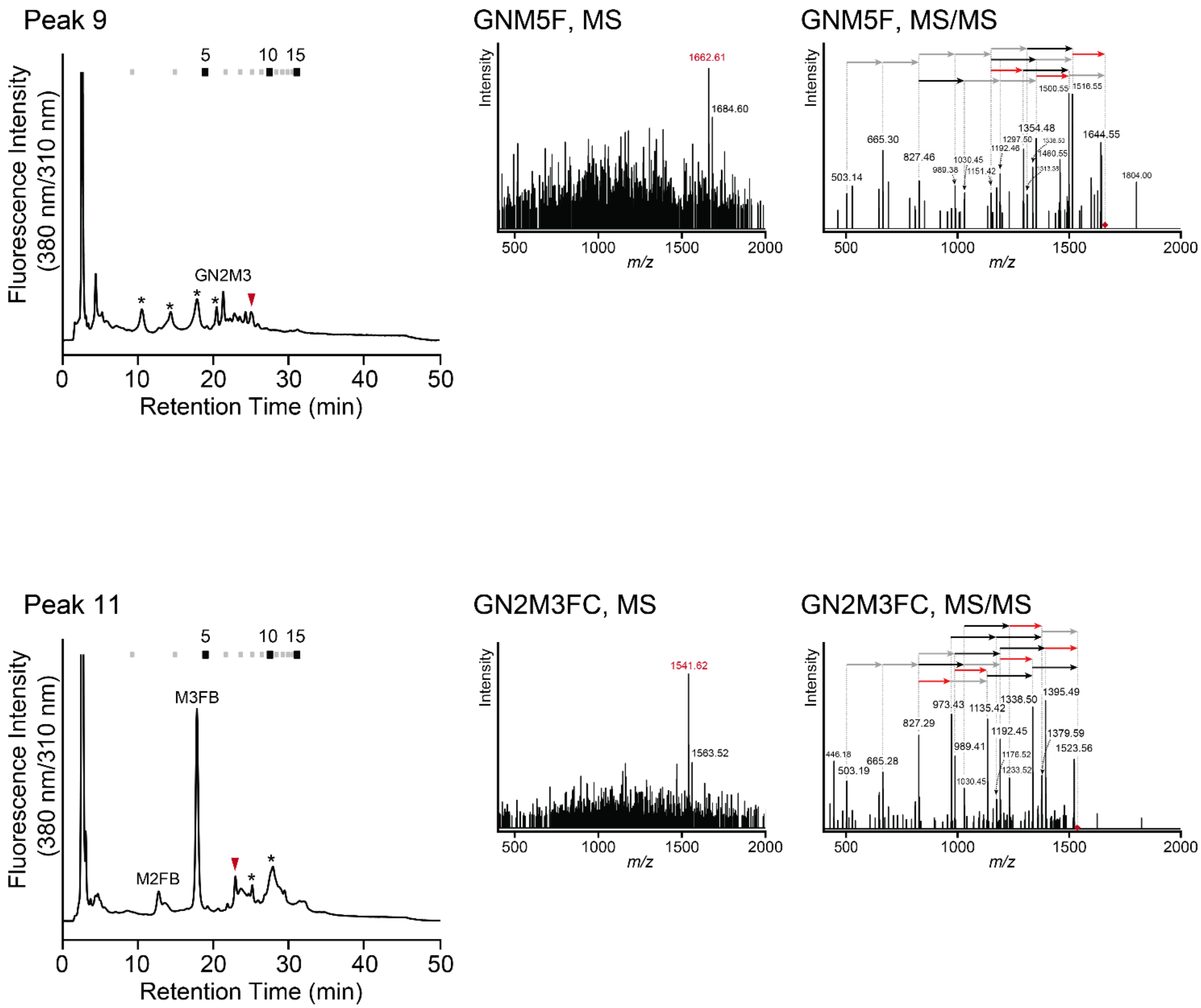

Supplementary Fig. S6 Structural analysis of *N*-glycans newly detected of hGNTIV expression

HPLC chromatograms and MS and MS/MS spectra of two *N*-glycans, GNM5F and GN2M3FC, which were not detected in WT glycan profiles. The symbols used in this figure are the same as those described in Supplementary Fig. 5.

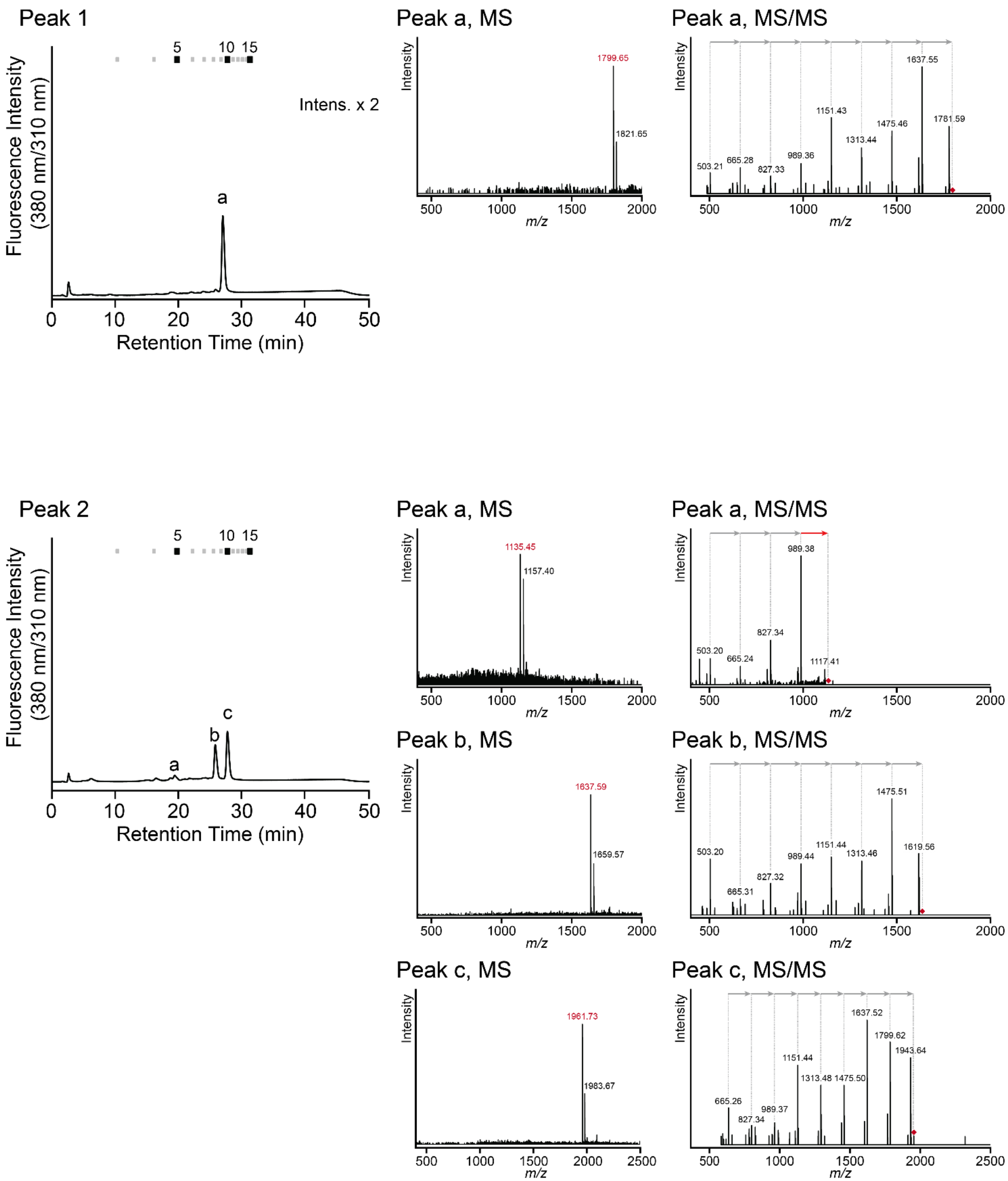

Supplementary Fig. S7 Structural analysis of *N*-glycans derived from cells expressing three genes, *bGNTI*, *bGNTII*, and *hGNTIV*.

Arrows, diamonds, and asterisks in the figure are defined as in Supplementary Fig. 5.

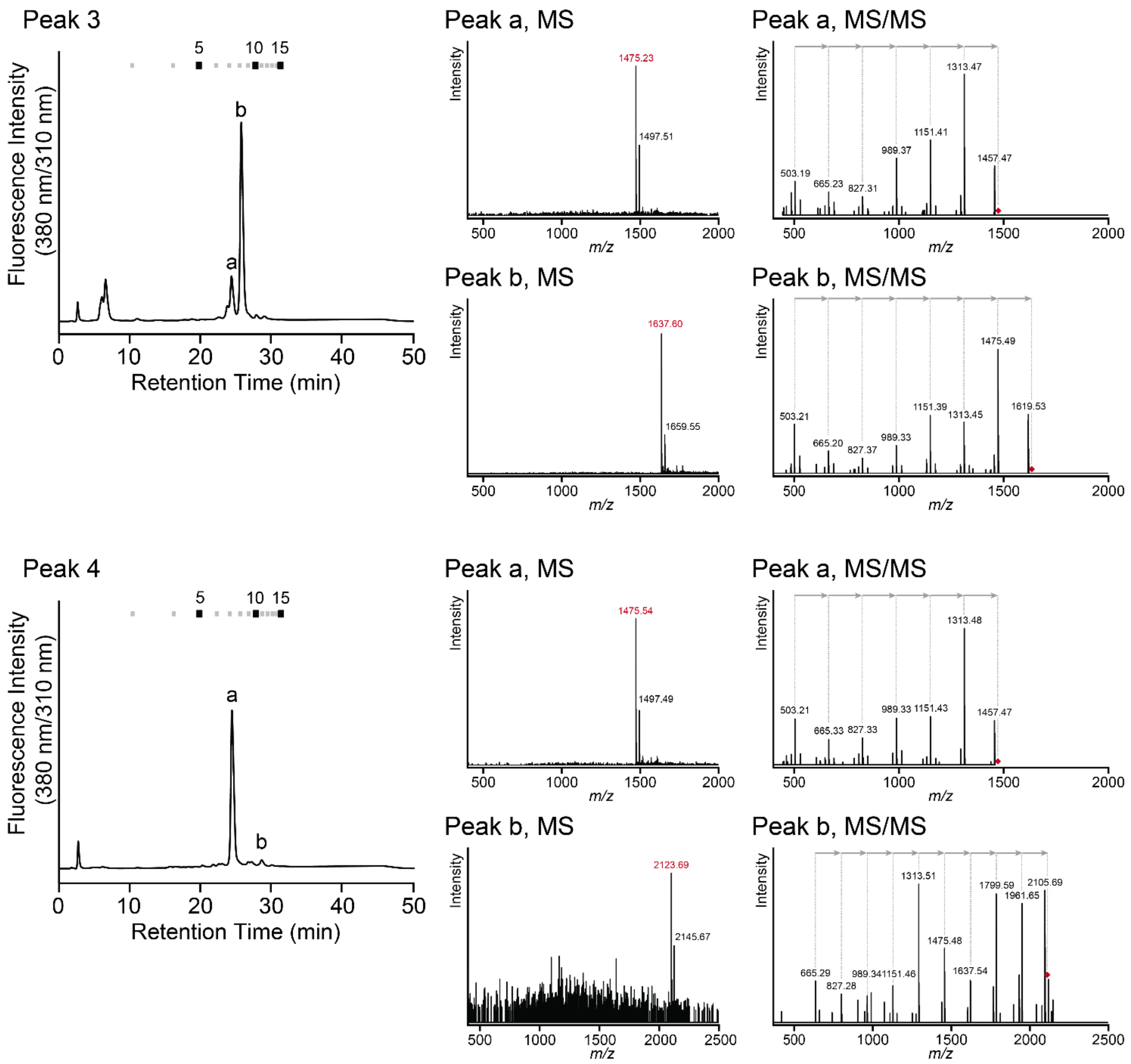

Supplementary Fig. S7 (continued)

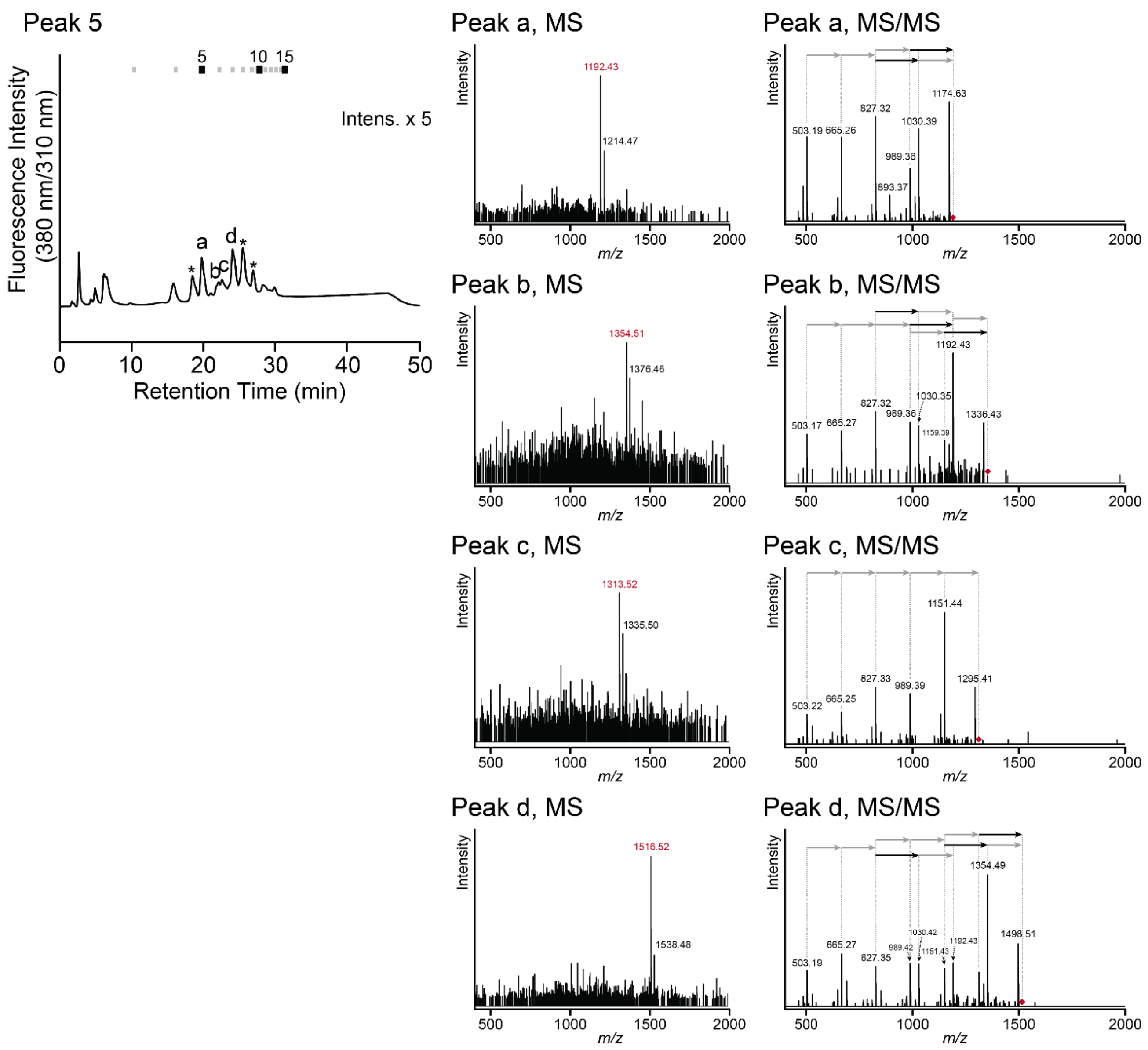

Supplementary Fig. S7 (continued)

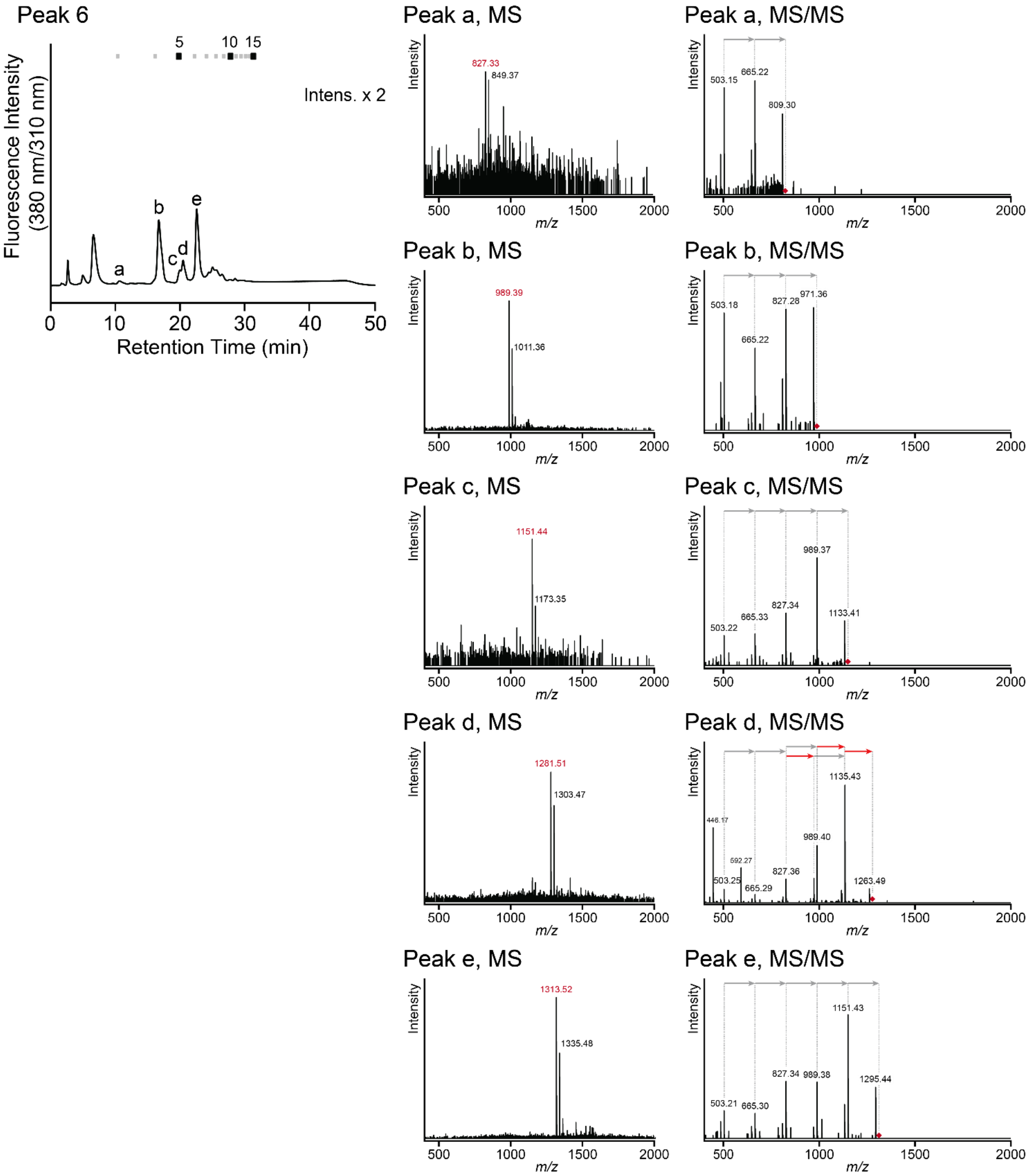

Supplementary Fig. S7 (continued)

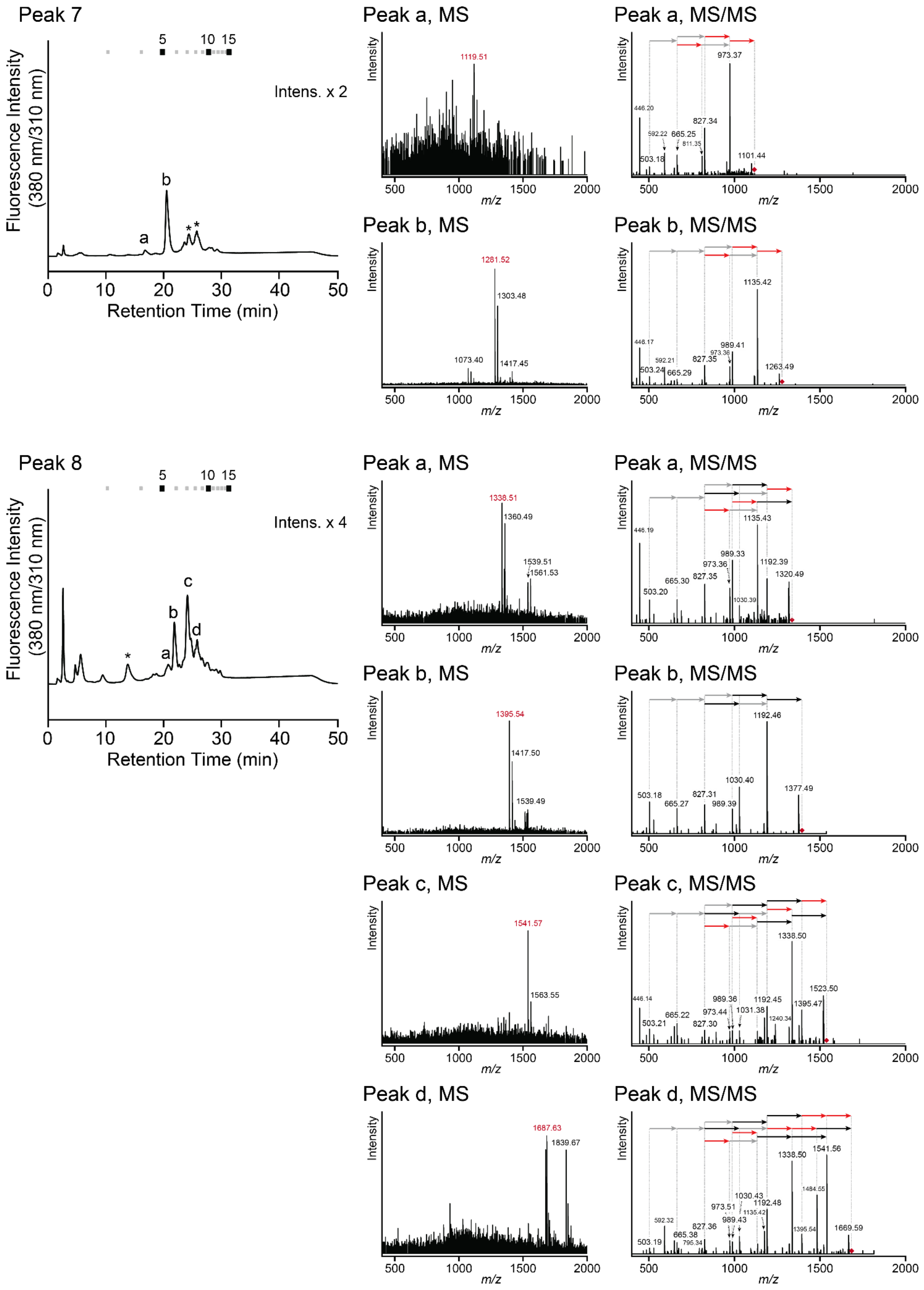

Supplementary Fig. S7 (continued)

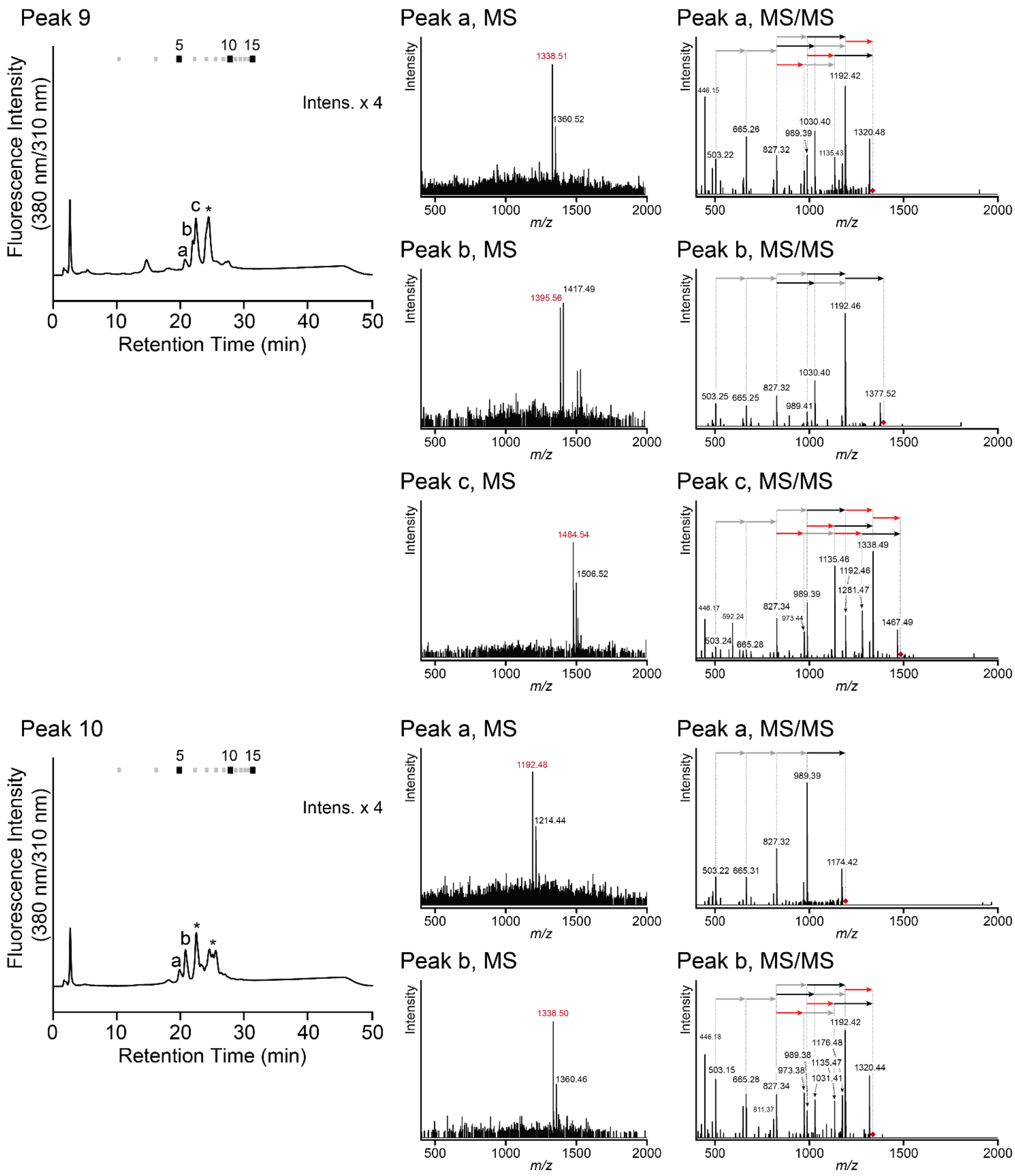

Supplementary Fig. S7 (continued)

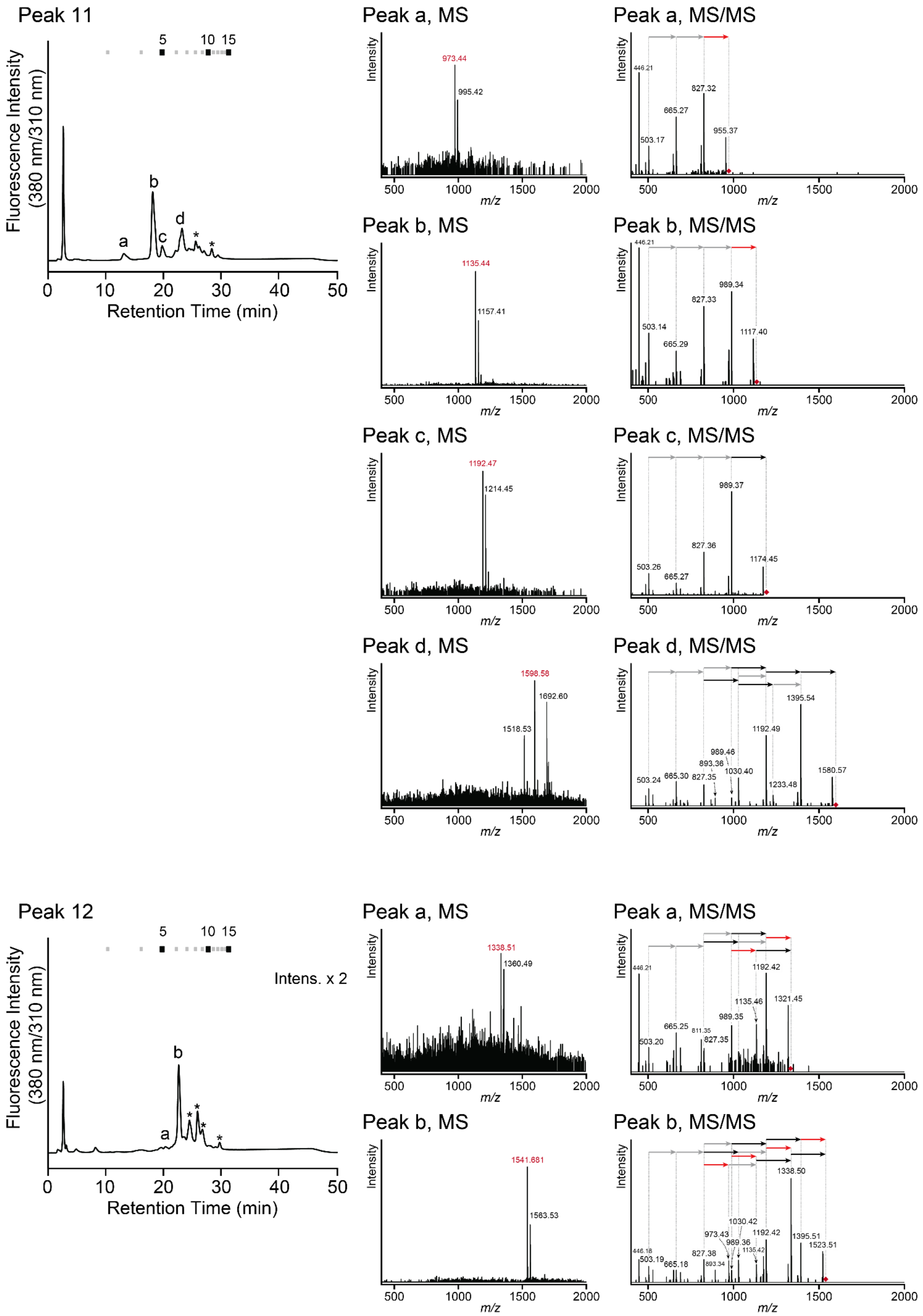

Supplementary Fig. S7 (continued)

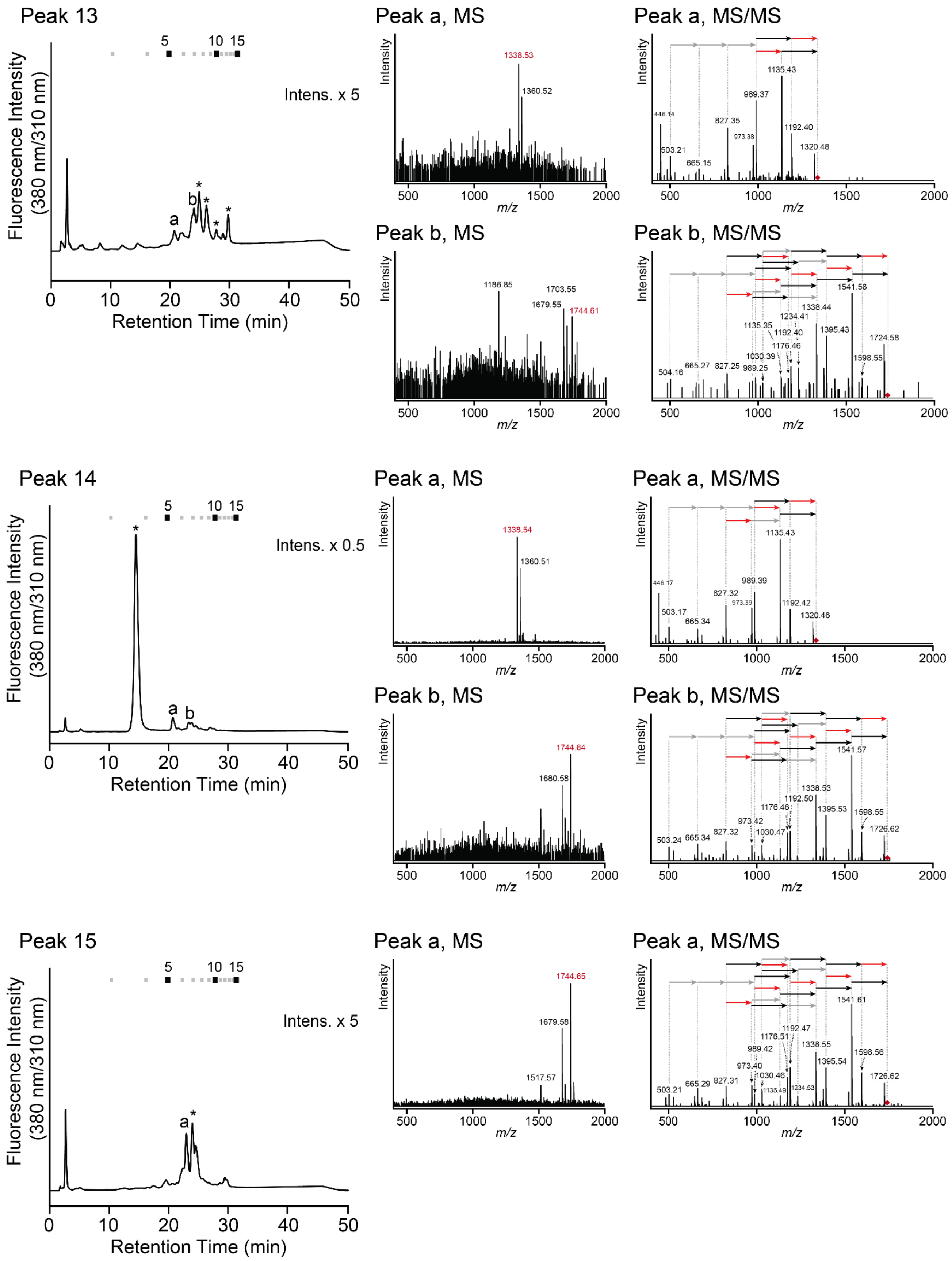

Supplementary Fig. S7 (continued)

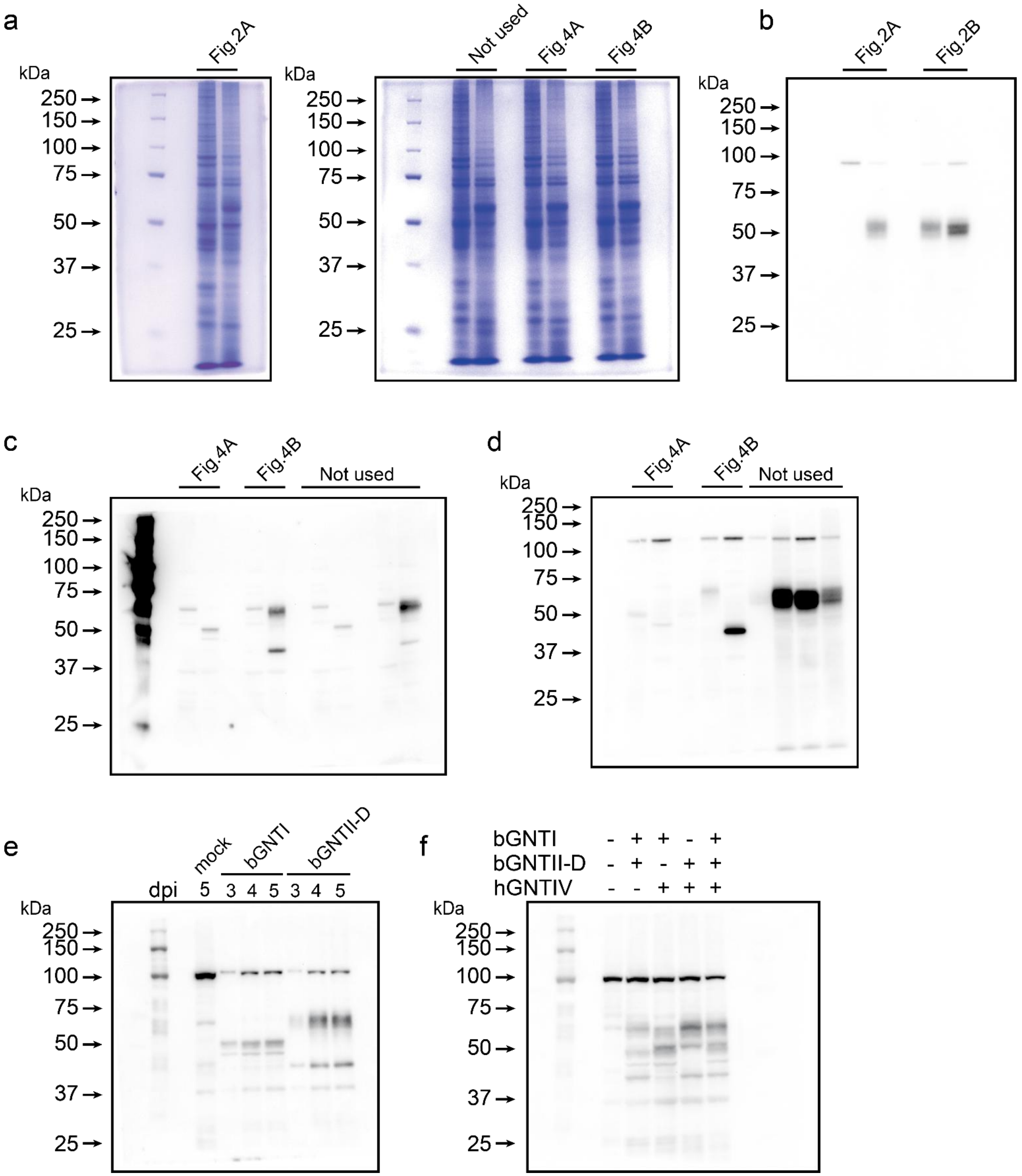

Supplementary Fig. S8. Uncropped images corresponding to (A) Fig. 2a and Fig. 4, (B) Fig. 2, (C) the Western blot in Fig. 4, (D) the PNGase F digestion in Fig. 4, (E) the Western blot in Fig. 6, and (F) Fig. 7.

Supplementary TABLE S1. Details of *N*-glycans detected in WT and hGNTIV-expressing Sf9 cells

Structure			Ratio (%)	
			WT	hGnTIV
**Mannose type *N*-glycan**				
		M3	6.2	5.4
		M4	-	0.6
		M5	4.6	6.8
		M6	10.1	11.0
		M7	20.9	19.5
		M8	18.8	18.8
		M9	4.7	6.0
		GlcM8	1.1	1.0
		GlcM9	1.7	1.2
**Fucosylated *N*-glycan**				
		M2FA	-	-
		M2FB	1.3	1.6
		M3FA	0.2	0.5
		M3FB	27.0	13.6
		M3FF	0.4	2.2
***N*-Acetylglucosaminylated *N*-glycan**				
	Mono *N*-acetylglucosaminylated *N*-glycan			
		GNM3A	0.7	1.1
		GNM3B	-	-
		GNM5	0.2	1.2
		GNM3FA	0.2	0.5
		GNM3FB	-	-
		GNM3FC	1.6	0.6
		GNM3FD	-	0.2
		GNM5F	-	0.2
	Bi-antenary *N*-glycan			
		GN2M3	-	1.5
		GN2M3FA	-	1.9
		GN2M3FB	-	3.0
		GN2M3FC	-	0.2
		GN2M3FF	-	0.1
	Tri-antenarry *N*-glycan			
		GN3M3	-	0.3
		GN3M3FA	-	0.1
		GN3M3FB	-	0.9
Total Mannose-type *N*-glycan			97.0	88.2
Total *N*-acetylglucosaminylated *N*-glycan			2.7	10.5
Total Tri-antennary *N*-glycan			-	1.3

The relative ratio of the structures was calculated on the basis of the peak area as determined LC-MS/MS analysis.

Supplementary TABLE S2 Relative amounts of *N*-glycans detected in bGNTI or bGNTII-D-expressing Sf9 cells

Structure		Ratio (%)	
		bGNTI	bGNTII-D
**Mannose type *N*-glycan**			
	M3	4.0	3.2
	M5	8.6	3.9
	M6	26.3	24.5
	M7	16.5	19.9
	M8	6.2	10.5
	M9	2.3	4.3
	GlcM8	0.3	-
	GlcM9	0.8	1.5
**Fucosylated *N*-glycan**			
	M2FA	0.2	-
	M2FB	0.9	-
	M3FA	0.3	0.3
	M3FB	9.3	7.2
	M3FF	7.1	4.1
***N*-Acetylglucosaminylated *N*-glycan**			
Mono *N*-acetylglucosaminylated *N*-glycan			
	GNM3A	2.1	0.6
	GNM3B	-	2.2
	GNM4	0.2	-
	GNM5	1.4	0.9
	GNM3FA	0.3	0.3
	GNM3FB	7.5	0.4
	GNM3FC	-	0.5
	GNM3FD	0.3	4.9
	GNM5F	5.2	-
	GNM3BFF	-	4.4
Bi-antenary *N*-glycan			
	GN2M3	-	1.7
	GN2M3F	-	1.8
	GN2M3FF	-	2.9
Total *N*-acetylglucosaminylated *N*-glycan		17.0	20.6
Total bGNTI product (GNM3A derivative)		9.2	10.8
Total bGNTII-D product (GN2M3 and GNM3B derivatives)		7.5	13.9

Supplementary TABLE S3 Temporal *N*-glycan changes in bGNTI or bGNTII-D-expressing Sf9 cells

Structure		Ratio (%)					
		bGNTI			bGNTII-D		
	dpi	3 dpi	4 dpi	5 dpi	3 dpi	4 dpi	5 dpi
**Mannose type *N*-glycan**							
	M3	8.4	3.6	5.9	4.7	4.2	3.9
	M4	-	0.3	0.4	0.3	0.4	0.4
	M5	10.0	8.1	9.3	6.5	7.9	8.0
	M6	21.8	23.4	17.0	14.1	21.7	20.8
	M7	9.4	18.3	9.6	23.9	17.6	18.7
	M8	6.9	6.1	5.1	10.1	7.8	7.4
	M9	3.0	1.8	2.5	7.1	4.0	3.8
	GlcM8	-	0.2	-	-	0.9	-
	GlcM9	-	0.9	0.6	0.9	-	0.6
**Fucosylated *N*-glycan**							
	M2FA	-	-	-	-	-	-
	M2FB	-	1.2	0.9	-	0.6	1.0
	M3FA	-	0.5	1.0	0.5	0.5	0.6
	M3FB	20.3	8.5	9.9	13.2	7.9	6.2
	M2FF	-	0.3	0.5	0.2	0.2	0.1
	M3FF	10.6	5.6	7.9	7.9	4.1	3.6
***N*-Acetylglucosaminylated *N*-glycan**							
Mono *N*-acetylglucosaminylated *N*-glycan							
	GNM3A	1.5	2.6	5.1	0.5	0.6	0.7
	GNM3B	-	-	-	0.8	2.7	3.0
	GNM4	-	0.3	0.5	-	-	-
	GNM5	1.2	1.9	3.3	0.4	0.5	0.5
	GNM3FA	0.1	0.4	0.6	0.1	0.4	0.4
	GNM3FB	-	0.7	0.7	0.2	0.5	0.5
	GNM3FC	3.5	8.7	14.5	1.2	0.6	0.3
	GNM3FD	0.9	-	-	1.6	5.3	5.3
	GNM5F	-	2.8	1.8	0.5	0.5	0.9
	GNM3BFF	-	0.8	0.9	3.2	3.2	4.9
Bi-antenary *N*-glycan							
	GN2M3	0.6	-	-	0.6	1.9	2.3
	GN2M3F	1.7	2.5	1.3	1.2	4.2	3.0
	GN2M3FF	-	0.7	0.6	0.3	2.2	3.0
Total *N*-acetylglucosaminylated *N*-glycan		9.5	21.4	29.3	10.6	22.6	24.8
Total bGNTI product (GNM3A derivative)		6.2	16.3	25.2	2.6	2.2	2.8
Total bGNTII-D product (GN2M3 and GNM3B derivatives)		3.3	5.1	4.1	8.0	20.4	22.0

Supplementary TABLE S4 Details of *N*-glycans detected in Sf9 cells expressing multiple *N*-acetylglucosaminyltransferases

Structure		Ratio (%)			
		bGNTI + bGNTII-D	bGNTI + hGNTIV	bGNTII-D + hGNTIV	bGNTI + bGNTII-D + hGNTIV
**Mannose type *N*-glycan**					
	M2	-	0.3	-	0.2
	M3	3.3	5.7	3.2	4.3
	M4	0.3	0.6	0.3	0.5
	M5	3.6	6.5	5.7	4.1
	M6	22.7	16.3	20.5	16.9
	M7	15.2	19.8	19.1	22.4
	M8	7.6	7.8	12.9	3.3
	M9	4.2	3.2	5.3	4.3
	GlcM8	-	0.3	0.2	-
	GlcM9	0.6	0.4	0.8	0.5
**Fucosylated *N*-glycan**					
	M2FA	-	-	-	0.6
	M2FB	-	1.3	0.4	1.2
	M3FA	0.5	1.0	0.3	0.5
	M3FB	10.8	10.2	8.9	8.5
	M5F	-	-	0.2	-
	M2FF	0.2	0.3	0.2	0.3
	M3FF	3.2	5.9	4.7	4.7
***N*-Acetylglucosaminylated *N*-glycan**					
Mono *N*-acetylglucosaminylated *N*-glycan					
	GNM3A	1.2	1.7	0.4	0.7
	GNM3B	3.0	0.9	2.0	1.8
	GNM4	0.3	0.2	-	0.2
	GNM5	1.5	1.3	0.2	1.0
	GNM3FA	-	0.1	0.2	-
	GNM3FB	0.4	0.2	0.3	0.3
	GNM3FC	1.3	4.8	0.5	0.9
	GNM3FD	3.7	0.5	2.9	3.1
	GNM5F	0.8	5.8	-	-
	GNM3BFF	2.8	-	1.5	1.3
Bi-antenary *N*-glycan					
	GN2M3	3.1	1.2	1.0	1.6
	GN2M3FA	0.1	-	-	-
	GN2M3FB	7.1	2.0	1.6	5.1
	GN2M3FC	-	0.7	-	-
	GN2M3FF	2.5	0.5	-	2.8
Tri-antenarry *N*-glycan					
	GN3M3	-	-	3.5	4.0
	GN3M3FA	-	-	-	0.1
	GN3M3FB	-	0.4	2.9	4.8
Total Mannose-type *N*-glycan		57.5	60.9	68.0	56.5
Total *N*-acetylglucosaminylated *N*-glycan		27.8	20.3	17.0	27.7
Total bGNTI product (GNM3A derivative)		5.1	13.9	1.3	2.8
Total bGNTII-D product (GN2M3 and GNM3B derivative		22.7	5.3	9.3	16.0
Total Tri-antennary *N*-glycan		-	0.4	6.4	8.9

Supplementary TABLE 5 Primer information used in this study

Primer	Squence (5' → 3')
M13_Fw	GTTTT CCCAG TCACG AC
M13_Rv	CAGGA AACAG CTATG AC
pFastBac_Fw	GGATT ATTCA TACCG TCCCA
pFastBac_Rv	CAAAT GTGGT ATGGC TGATT
Bmrp49-Fw	CAGGC GGTTC AAGGG TCAAT AC
Bmrp49-Re	TGCTG GGGCT CTTTC CACGA
EcoRI-kozac-BmGnTI_Fw	TATGA ATTCG CCATG CGCCT GAATG
HindIII-S-6HisBmGnTI_Rv	TATAA GCTTC TAGTG ATGGT GATGG TGATG GCCCC ACGAG
BamHI-kozac-TM-BmGnTII_Fw	TATGG ATCCG CCATG CCACG GCTCA AGTAT GCTTC GGTCG TGCGT GTTGT CGTCA
HindIII-S-6HisBmGnTII_Rv	TATAA GCTTC TAGTG ATGGT GATGG TGATG AGCGT AGTAA TT
